# Extending the Monitoring of Perfluoroalkyl Substances in Arctic Air Reveals a High Abundance of Both Short Acids and Neutral Compounds

**DOI:** 10.1021/acs.est.5c05145

**Published:** 2025-08-01

**Authors:** Alexander Kasperkiewicz, Frank Wania, Fiona Wong, Alexander Vlasenko, Henrik Li, Jared Chisamore, Helena Dryfhout-Clark, Phil Fellin, Hayley Hung

**Affiliations:** † Air Quality Processes Research Section, 6347 Environment and Climate Change Canada, 4905 Dufferin Street, Toronto, Ontario M3H 5T4, Canada; ‡ Department of Chemical Engineering and Applied Chemistry, 33530 University of Toronto, Toronto, Ontario M5S 3E5, Canada; § 383790 Airzone One Ltd, 222 Matheson Blvd. E., Mississauga, Ontario L4Z 1X1, Canada; ∥ Department of Physical and Environmental Sciences, University of Toronto Scarborough, Toronto, Ontario M1C 1A4, Canada

**Keywords:** active air sampling, arctic, long-range atmospheric transport, short-chain PFAS, method development

## Abstract

Interest in per- and polyfluoroalkyl substances (PFASs) in the remote atmosphere now extends to perfluoroalkyl carboxylic acids (PFCAs) and perfluoroalkyl sulfonic acids (PFSAs) with short (*n*_C_ < 4), medium (3 < *n*_C_ < 13), and long (*n*_C_ > 12) alkyl chains. A liquid chromatography–mass spectrometry method for the combined analysis of PFASs of variable chain length was applied to 204 high volume active air samples collected at Alert, Nunavut (82° 30′ N 62° 20′ W) between March 2014 and October 2023. Short-chain PFASs (scPFASs) were detected frequently (>75%) and at the highest median concentrations (trifluoroacetic acid (TFA): 20 pg/m^3^, perfluoropropionic acid (PFPrA): 1.1 pg/m^3^, perfluorobutanoic acid (PFBA): 3.7 pg/m^3^), while n_C_ > 10 PFAS were sparsely detected (detection frequency [DF] < 20%). Using a suspect-screening approach, hexafluoro-2,2-propanediol (HF2OH) and hexafluoroisopropanol (HFIPA) were confirmed in Arctic air at DF exceeding 75%. We find that concentrations of TFA and HF2OH were significantly correlated with temperature and increased during snowmelt periods, suggesting local emission or precursor release followed by degradation processes. The modified OECD LRTP and Pov assessment tool supported the potential of HFIPA and HF2OH to undergo long-range atmospheric transport. Time trend analysis reveals that after a short period of stable or declining levels in the mid-2010s, concentrations of PFBA, PFOA, and PFOS in Arctic air are increasing again since 2019, which may be a useful consideration when evaluating the effectiveness of the Stockholm Convention’s listing of PFOA and PFOS.

## Graphical abstract

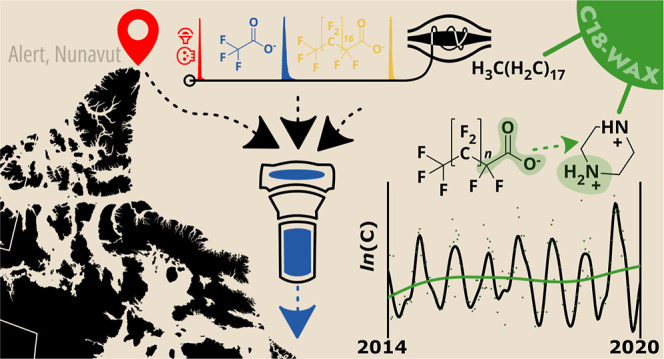

## Introduction

Significant environmental and regulatory attention has been given to select compounds of the per- and polyfluoroalkyl substance (PFAS) class, namely, medium (3 < *n*_C_ < 13) and long (*n*_C_ > 12) alkyl chains. The short-chain members (*n*_C_ < 4, scPFASs) of this vast chemical group have, until recently, not been the focus of regulatory scrutiny due to previously suspected lower bioaccumulation potential, although their persistence is comparable. They have been reported in drinking water, soils, seawater, glacial ice, and air and are thought to be ubiquitous anthropogenic contaminants in the environment.^1–5^ Trifluoroacetic acid (TFA) concentration levels have been reported to be increasing in many matrices and locations since the 1990s.^3,6–8^ Increasing environmental concentrations of TFA, perfluoropropanoic acid (PFPrA), and perfluorobutanoic acid (PFBA) are attributed to the breakdown of new-generation fluorinated refrigerants such as hydrofluorolefins, hydrofluorocarbons, and hydrofluoroethers.^9^ In the pursuit of reducing the stratospheric ozone depletion and global warming potential of halogenated refrigerants, their replacements contribute to increasing scPFAS levels globally.^10–13^ The scientific community remains divided on whether the observed increases in TFA concentrations represent a sustainable trajectory, with cases made that current accumulation rates may exceed ecological thresholds,^14^ while others maintain that environmental systems can accommodate increased levels.^11,15^

Due to their persistence and high mobility, scPFASs and their precursors are susceptible to long-range environmental transport (LRET). However, limited data exist about their air concentration levels in remote regions. Initiated to fill the knowledge gap of air concentrations of persistent organic pollutants (POPs) and Chemicals of Emerging Arctic Concern (CEAC), including PFAS, a national long-term monitoring program at Alert, NU, Canada, has been in operation since the 1990s.^3,16–18^ The program has provided information on remote air concentrations of POPs and CEAC via routine high-volume active air sample collection (HV-AAS) and can be leveraged to provide information on air concentrations of scPFAS in the High Arctic.^19^

A common analytical approach for the analysis of PFAS involves the application of three instrumental methods: a gas chromatography–mass spectrometry (GCMS) method for neutral PFASs such as the fluorotelomer alcohols (FTOHs) and acrylates (FTACs),^20^ a reverse-phase liquid chromatography–mass spectrometry (LCMS) method for medium- and long-chain PFCAs, PFSAs, and other groups,^21^ and a hydrophilic interaction (HILIC),^1^ ion-exchange,^3^ or supercritical fluid (SFC)^17^ MS run for scPFAS such as TFA and PFPrA. Due to the significant workload required to gather a holistic data set on a sample’s PFAS content, efforts have been made to improve the efficiency of analytical approaches. These efforts include the derivatization of perfluorocarboxylic acids (PFCAs) to make them amenable to GCMS analysis^22^ or differing mobile phase additives enabling FTOH and FTAC analysis via LCMS.^23^ Also, the availability of mixed-mode stationary phases containing both nonpolar and weak-anion exchange (WAX) functionality allows for the improved retention of scPFASs while maintaining separating capability for medium- and long-chain PFASs via reverse-phase mechanisms.^24^

In this work, we present a method leveraging a mixed-mode column to enable the simultaneous analysis of 1 < *n*_C_ < 19 PFCAs along with select perfluorosulfonic acids, perfluorosulfonamides, and hexafluoropropylene oxide dimer acid (HFPO–DA), for a total of 26 targeted compounds. Following method validation, the approach was applied to the analysis of air samples collected at Alert, NU, Canada, over a ten year period. The use of high-resolution mass spectrometry (HRMS) instrumentation allowed for interrogation via a suspect-screening approach focused on related scPFAS, resulting in the confirmation and measurement of hexafluoro-2,2-propanediol (HF2OH) and hexafluoroisopropanol (HFIPA). The objectives of this work were to develop an analytical approach to incorporate scPFAS as part of medium- and long-chain PFAS analysis, characterize long-term and seasonal temporal trends of scPFAS in Arctic air, and identify potential source processes through correlation analysis with air temperature as well as air concentration level changes during and following snowpack melt events.

## Materials and Methods

### Reagents and Materials

LC/MS grade methanol (MeOH) and water were obtained from Fisher Scientific (Hampton, NJ, USA). LC/MS-grade acetic acid and ammonium acetate were obtained from Sigma-Aldrich (St. Louis, MO, USA). Standards of TFA and PFPrA along with labeled ^13^C_2_-TFA and ^13^C_3_–PFPrA were from Cambridge Isotope Laboratories, Inc. (Andover, MA, USA). Standards of PFBA, perfluoropentanoic acid (PFPeA), perfluorohexanoic acid (PFHxA), perfluoroheptanoic acid (PFHpA), perfluorooctanoic acid (PFOA), perfluorononanoic acid (PFNA), perfluorodecanoic acid (PFDA), perfluoroundecanoic acid (PFUnDA), perfluorododecanoic acid (PFDoDA), perfluorotridecanoic acid (PFTrDA), perfluorotetradecanoic acid (PFTeDA), perfluorohexadecanoic acid (PFHxDA), perfluorooctadecanoic acid (PFODA), perfluorobutanesulfonic acid (PFBS), perfluorohexanesulfonic acid (PFHxS), perfluorooctanesulfonic acid (PFOS), perfluorodecanesulfonic acid (PFDS), hexafluoropropylene oxide dimer acid (HFPO–DA), perfluorobutane sulfonamide (FBSA), perfluorohexane sulfonamide (FHxSA), perfluoroheptane sulfonamide (FHpSA), perfluorooctane sulfonamide (FOSA), perfluorodecane sulfonamide (FDSA), and perfluoro-4-ethylcyclohexanesulfonate (PFECHS) were from Wellington Laboratories (Guelph, ON, Canada). Isotopically labeled compounds ^18^O_2_–PFHxS, ^13^C_4_–PFOS, ^13^C_4_–PFOA, ^13^C_4_–PFBA, ^13^C_5_–PFNA, ^13^C_2_–PFHxA, ^13^C_2_–PFDA̧^13^C_2_–PFUnDA̧^13^C_2_–PFDoDA̧ ^13^C_8_–FOSA, ^13^C_8_–PFOS, and ^13^C_8_–PFOA were also from Wellington Laboratories. Hexafluoroacetone (HFA) and hexafluoroisopropanol (HFIPA) were from Sigma-Aldrich.

### Sampling Methods and Sample Preparation

Sampling methods and location details have been described in a previous work.^16^ Briefly, air sampling was conducted at the Global Atmospheric Watch Laboratory at Alert, Nunavut, Canada, (82° 30′ N, 62° 20′ W) between March 2014 and October 2023. The 204 samples were collected using a Tisch PS-1 HV-AAS, with each sample representing approximately 2000 m^3^ of air collected over 7 days. Sampling occurred monthly from October–February and biweekly from March–September. Samples were collected using a glass fiber filter and a sorbent pack consisting of two polyurethane foam (PUF) plugs (Tisch Environmental, Inc., Cleves, OH, USA) and 5 g of XAD-2 resin (Amberlite XAD-2, Sigma-Aldrich). Full sample preparation details are presented in S1 and in prior work.^16^ Across the whole sample set, samples were extracted using accelerated solvent extraction (ASE 200, Dionex Corp., Sunnyvale, CA, USA). An internal standard (IS) mixture was added to the samples prior to extraction, and an injection standard mixture was added prior to instrumental analysis. For all compounds, except for TFA, PFPrA, HF2OH, and HFIPA, the isotope dilution method was used for quantification. For TFA, PFPrA, HF2OH, and HFIPA, semiquantitation was performed using injection standards only (^13^C_2_-TFA and ^13^C_3_–PFPrA). Breakthrough was evaluated by extracting one summer and one winter sample, both taken in 2023, in two fractions, with the top PUF and the XAD-resin extracted separately from the bottom PUF.

### Instrumental Analysis and Optimization

To modulate the WAX moieties on the stationary phase, a mobile phase composition enabling a pH gradient must be used. We opted for a gradient of water with 0.025% acetic acid and MeOH with 20 mM ammonium acetate, shared in the SI S3, with a chromatogram of the separation of select compounds in the target panel shown in the SI (Figure S1). Due to the fragility of PFCAs to in-source fragmentation, and their increased susceptibility with reduced chain length,^25^ a Box-Behnken experimental design was used to investigate optimal ion source conditions for the molecular ion signal of scPFAS, with hopes of minimal compromise to instrumental sensitivity for longer-chain targets.

The optimized instrumental method details are available in part S3 in the SI. In brief, air samples were analyzed using a Thermo Scientific Vanquish system (Thermo Fisher Scientific, Waltham, USA) coupled to a Thermo Scientific Orbitrap Exploris 240 mass spectrometer. The chromatographic separation was completed using a Waters Atlantis Premier BEH C18 AX column (1.7 μm, 2.1 × 50 mm, Waters Corporation, Milford, USA) equipped with a Waters ACQUITY UPLC BEH C18 VanGuard Precolumn (1.7 μm, 2.1 mm × 5 mm) as well as a Waters Isolator Column as a delay column. A 3-step, 15 min gradient method using the aforementioned mobile phases was run at a flow rate of 400 μL/min, a column temperature of 45 °C, and an injection volume of 4 μL. Samples were not reconstituted, only blown down to 0.5 mL in MeOH prior to injection. The mass spectrometer was operated in full scan, data-dependent acquisition (ddMS^2^), and data-independent acquisition (DIA) modes, with full parameters of targeted masses, filter tree details, and optimized ion source parameters located in the SI (Table S4). Ion source parameters were optimized for the total signal observed from in-source fragment and parent molecule of 1 < *n*_C_ < 6 PFCAs and HFPO–DA using a Box-Behnken experimental design (4 center points per block), with electrospray voltage (2000 to 3500 V), ion transfer tube temperature (200 to 350 °C), and vaporizer temperature (200 to 400 °C) as continuous factors. Additional information on the design of experiments can be found in S4.

### Quality Assurance and Data Processing Details

Data included in this work are produced by the Organics Analysis Lab, which is a participant of the Arctic Monitoring and Assessment Programme/Northern Contaminants Program (AMAP/NCP) interlaboratory studies, details of which can be found in prior work.^16^ At every third sampling period, a field blank was collected by installing sampling cartridges on the HV-AAS and leaving the GFF surface exposed for 1 min, before storage, transport, and processing with samples. Additionally, a laboratory blank was taken for every batch undergoing sample extraction. Along with the 204 samples, 68 field blanks and 32 laboratory blanks were analyzed with this data set. Data was processed using Thermo Scientific TraceFinder 5.2 SP3, and select samples were further processed via a suspect screening approach using Thermo Scientific CompoundDiscoverer 3.3 SP3, with workflow settings shared in S6 (Table S15). The Box-Behnken design of experiment (DoE) was developed and processed using an OriginPro 2024b (OriginLab Corporation, Northampton, USA). Information on the limit of detection (LOD), method detection limits (MDLs), the analytical figures of merit for the compound panel, and the method of recovery calculation can be found in S5. The digital filtration technique used to derive time trends and seasonal cycles of selected chemicals is described in S10; these methods have been used previously.^16,26^ Spearman correlations between air temperature during sampling were calculated using OriginPro2024b, with additional details in S9. Analysis of snowpack melt events and significant differences between air concentrations of PFAS and snowpack melt progress were completed using Kruskal–Wallis Analysis of Variance (ANOVA) with Dunn’s test used for posthoc analysis using R and the dunn.test R package.^27^

### Data Reporting and Literature Comparisons

The atmospheric measurements of PFSAs, PFCAs, and related fluorinated strong acids via HV-AAS employing a fiber filter followed by a sorbent pack have been reported to overestimate the particle-bound fraction of these compounds.^28–31^ Artifacts caused by blow-on and sorption to filters^32,33^ result in gas/particle partitioning data, which cannot be compared to samples collected using gas-phase passive, denuder, or online sampling techniques.^34,35^ Total gas- and particle-phase concentrations have been shown to differ by less than a factor of 2 between HV-AAS and denuders for 3 < n_C_ < 9 PFCAs and PFSAs.^35^ Thus, data are reported here as the summed concentration of analytes quantified in the extracts of both the GFF and the PUF/XAD/PUF cartridge.

### Evaluation of Long-Range Transport Potential and Snowpack Partitioning

The modified OECD tool (Version 0.99–21.06.2022) by Breivik et al.^36,37^ with default environmental input parameters and a cold receptor region was used to estimate the LRET potential of HF2OH and HFIPA. The chemical input parameters as well as results for the two chemicals are shown in S7. Chemical partitioning behavior in Arctic snowpack was evaluated using a similar approach to that described in Meyer et al.^38^ Further details along with relevant chemical properties are given in S8.

## Results and Discussion

### Instrumental Method Optimization and Performance Characteristics

The results of the ion source optimization are presented in detail in S4. Briefly, two distinct behavioral patterns were observed. For scPFCAs and HFPO–DA, transfer line temperature emerged as the primary significant effect, with operation at 200 °C yielding 3–10 times higher normalized areas compared to 350 °C (Figures S2 and S3). In contrast, sulfonic acids and sulfonamides showed less sensitivity to transfer line temperature, with vaporization temperature as the dominant factor affecting their normalized areas (range of 0.77–0.99 across the 250–400 °C response surface for PFHxS). The gains in relative area made via lower transfer line temperature for scPFCAs (3-fold for TFA, PFPrA) outweighed the marginal decrease observed for PFSAs between optimum at 275 and 200 °C (1.1-fold difference for PFHxS). Based on these findings, the optimal ion source parameters were established at 200 °C transfer line temperature, 325 °C vaporization temperature, and −3000 V electrospray voltage to maximize molecular ion detection of fragmentation-prone compounds.

Analytical figures of merit for the method are listed in Table S7. The instrumental LODs for most medium-chain PFCAs and PFSAs (0.0033–0.025 pg/m^3^, 53–400 fg on column) are within an order of magnitude of those of previously reported analytical methods for air (0.0063–0.031 pg/m^3^)^16^ and water (25–1250 fg)^1^ (Table S7). The detection limits of 4000 fg for TFA and 2000 fg for PFPrA reported here are also within an order of magnitude of earlier HILIC and SFC approaches.^1,8,17^ However, our method cannot match the impressively low detection limits of 55 fg for TFA and 156 fg for PFPrA that have been achieved using derivatization followed by GCMS.^22^ Additional comparisons are shown in Table S8 in the S I. We note, due to the Orbitrap analyzer used in this work, higher absolute LODs can be expected for large compounds, such as PFODA and PFHxDA, when operated in full scan mode across a broad mass range as reported (80 −1000 *m*/*z*), which is a compromise of the method developed.^39,40^

### Confirmation of Hexafluoro-2,2-Propanediol and Hexafluoroisopropanol in Arctic Air

Hexafluoro-2,2-propanediol (HF2OH) and HFIPA were confirmed to a level of 1b using analytical standards, with matching criteria for HF2OH and HFIPA shown in Figures 1 and S4, respectively.^41^ HF2OH is the gem-diol form of HFA, which is expected to be hydrated in the environment due to the high hydration constant (log *K*_hyd_ = 6.08) of the carbonyl imposed by fluorination (as shown in the inset of Figure 1).^42,43^ The conversion to the gem-diol has been modeled to be energetically favorable on ice surfaces.^44^ HFA is used in the synthesis of HFIPA,^45^ whereas HFIPA is used in a variety of organic synthesis applications,^46^ notably as a precursor in the industrial synthesis of sevoflurane, an inhaled medication used to induce and maintain general anesthesia. It is also a human metabolic product of sevoflurane.^47,48^ Reports of the environmental occurrence of HFIPA and HFA/HF2OH are rare. High HFIPA concentrations in contaminated soil around oil processing and electronic waste production facilities (with maximum concentrations of 657 ng/g and 854 ng/g, respectively) have been reported.^2,49^ HFIPA has been measured at concentrations up to 0.4 μg/L in 3 of 46 samples of German drinking water.^1^ HF2OH is rarely reported in the environment, with limited reports of HFA in industrial effluent dating to the 1970s,^50,51^ along with a recent level 2 identification of HF2OH in soil collected from an oil refinery.^2^ To the best of our knowledge, neither HFIPA nor HF2OH has been reported in any environmental compartment in remote regions to date.

**Figure 1:**
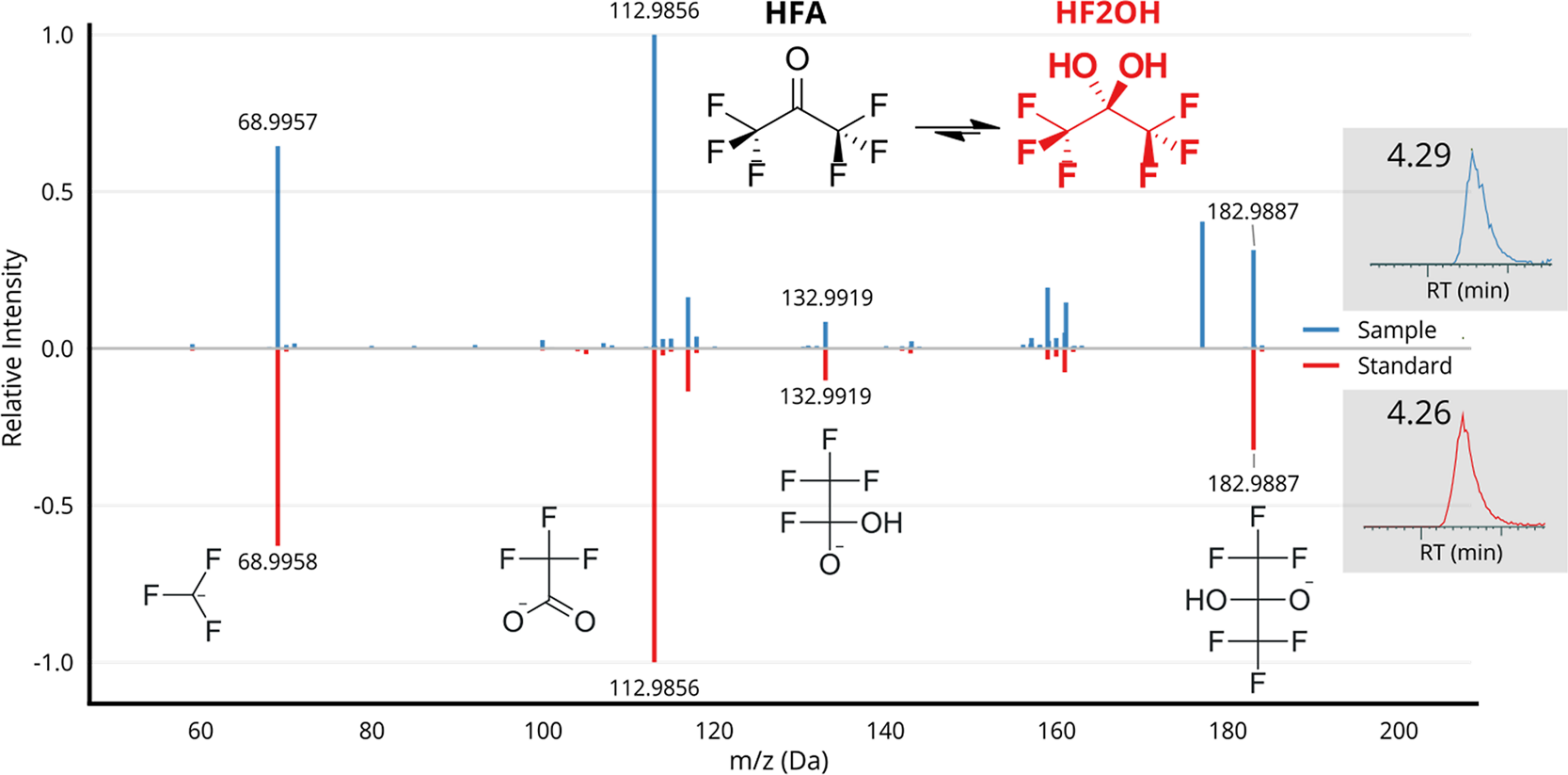
Presence of HF2OH was confirmed in samples at level 1b confidence, with excellent match between sample and standard for both HRMS fragmentation and retention time (equivalent plot for HFIPA in Figure S4).

Results of the updated screening tool for persistence and LRET potential^36^ for HFIPA and HF2OH are shown in Figure S5. High calculated fractions subject to dispersion (ϕ_1_), transfer (ϕ_2_), and accumulation in cold remote surface media (ϕ_3_) for both HFIPA and HF2OH suggest that they have a high potential for atmospheric LRET. For both HFIPA and HF2OH, emissions into air result in the largest fractions being dispersed and advected into cold, remote regions such as the Arctic, exceeding POP-like thresholds. These modeling results align with our field observations, suggesting that LRET is likely responsible for the presence of HF2OH and HFIPA in the Arctic. While the modeled LRET potential is not always consistent with empirical evidence,^52^ here, the combination of modeling predictions and empirical field measurements provides complementary evidence supporting the propensity of these compounds for LRET.

### Short-, Medium-, and Long-Chain PFAS in Arctic Air

Although TFA and PFPrA were corrected via postextraction labeled standards, external recovery experiments demonstrated that extraction accuracy and precision (% RSD) for these compounds were acceptable (109.5 ± 5.2/113.5 ± 2.3% for TFA extracted from filter/sorbent pack, 102.3 ± 6.1/105.8 ± 3.4% for PFPrA extracted from filter/sorbent pack, Table S10). Also, breakthrough analysis of the sorbent pack resulted in <14% breakthrough for both compounds (Table S9), comparable to breakthrough measured for PFOA. Quantitative analytical figures of merit for HF2OH and HFIPA (Table S10) were poorer. Specifically, extraction method recovery for HF2OH was 60% (RSD = 5.8%, *n* = 3) with breakthrough of 7.6 and 31% in samples taken during winter and summer, respectively. HFIPA proved even more quantitatively elusive, with a recovery of only 32% (RSD = 4.4%, *n* = 3). Its breakthrough could not be characterized because HFIPA was not detected in the two breakthrough samples. Two factors likely contributed to the low recoveries compared to the rest of the PFASs. The samples were extracted in two fractions, with the first hexane fraction likely partially extracting the neutral compounds. Also, both species are susceptible to evaporation loss during sample concentration, although this loss is predicted to be less thermodynamically favored from MeOH compared to hexane, with HFIPA predicted to be 3 orders of magnitude less volatile from MeOH (log *K*_Methanol‑Air_ = 4.76 vs log *K*_Hexane‑Air_ = 1.7, Abraham Absolv).^53^

In Alert air samples, scPFAS had both higher concentrations and detection frequencies when compared with medium- and long-chain PFAS, with 81–95% of samples above MDLs (Figure 2). TFA showed the highest concentrations (mean: 37.3, median: 19.6, range 251–1.76 pg/m^3^), followed by PFBA (mean: 4.31, median: 3.71, range 21.4–0.06 pg/m^3^) and PFPrA (mean: 1.31, median: 1.09, range: 10.8–0.134 pg/m^3^). To the best of our knowledge, no measurements of air concentrations of TFA and PFPrA have been reported in remote polar regions. The mean TFA concentration for this sample set was 20-fold lower than measurements made in Guelph, Ontario, Canada (760 pg/m^3^) in 2000 and 30-fold lower than those in 2015–16 in Beijing, China (1162 pg/m^3^).^6,54^ Similarly, PFPrA air concentration ranges of 64–360 pg/m^3^ measured via passive samplers in 2016 in Tianjin, China were 30 to 400 times greater than those in this work. HF2OH and HFIPA were detected with high frequency (>75%) in the analyzed air samples. Nominally, HFIPA was measured at a median air concentration and DF of 4.5 pg/m^3^ and 77%, whereas those numbers for HF2OH were 0.79 pg/m^3^ and 87%. These air concentrations should be treated as semiquantitative due to relatively low recoveries and uncertain breakthrough discussed above. Long-chain PFASs such as PFTrDA, PFTeDA, PFHxDA, and PFODA were detected less frequently (1–6% above MDL). HFPO–DA was recently reported in Arctic snow^21^ but was not detected in any sample in this work above MDL, potentially due to in-source fragmentation leading to a relatively high LOD (0.0625 pg/m^3^) compared to that of other PFCAs (0.0125 pg/m^3^ for PFHxA).

**Figure 2:**
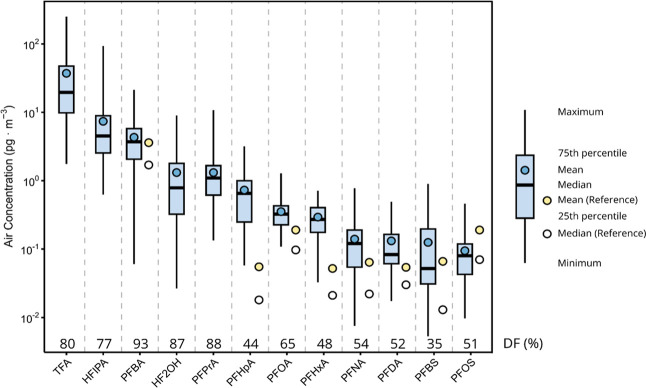
Box-plots of the concentrations (sum of particle filter and gas phase sorbent) of the ten PFASs most frequently detected in Canadian Arctic Air (2014–2023). Compounds have been ordered by arithmetic mean concentration. Numerical detection frequencies are reported as the percentage of 204 samples with levels above the MDL. Yellow and white points indicate the mean and median of the 2006–2014 subset of the current data set from Wong et al. 2018, as a comparison.

### Temporal Trends

The medium- and long-chain PFAS analytes have been monitored at Alert, NU, since 2006, with trends in total air concentrations reported previously.^16,19^ Updated trends for PFOA, PFOS, and PFBA are shared in S10 and in Figure 3. Several of the PFASs display trends where a period of rapid increase up to 2013 was followed by a period of temporary decline or stabilization only to increase again more recently. The start of that most recent increase occurred earlier for PFBA (2017) than for PFOA and PFOS (2019). In the case of PFBA and PFOA, concentrations recorded in 2023 have almost reached again the peak levels of 2013, whereas those of PFOS remain 20-fold lower than those a decade earlier. The PFOA time trend may reflect a transition occurring throughout the 2010s when releases mostly in the United States and Europe were replaced by releases in China.^55^ Also, emissions of PFOS have been decreasing since the 1990s, which is supported by recent atmospheric deposition rates determined in glacial ice cores for the period of 1967–2016.^56^ Despite PFOS and PFOA being listed under the Stockholm Convention as Persistent Organic Pollutants (in 2009 and 2020, respectively), the trend reversals observed in this study may be useful as a consideration in the effectiveness evaluation of the Convention. This behavior aligns with an increasing number of regulated POPs whose air concentration levels remain unaffected by their Stockholm Convention listing dates.^57^

**Figure 3:**
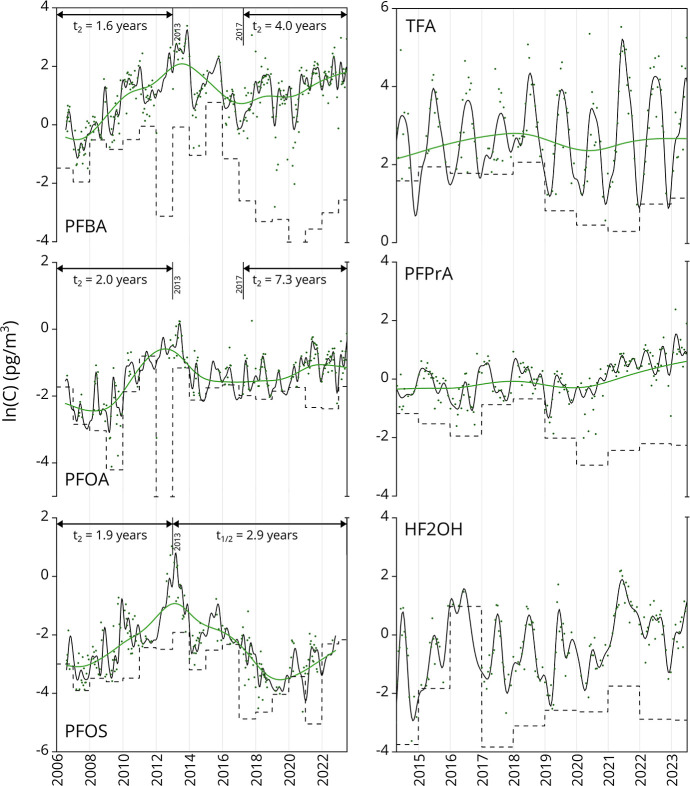
Long-term trends for select PFAS. Trends (green solid line) and seasonality (black solid line) across 2014–2023 for TFA, PFPrA, and HF2OH, along with 2006–2023 for PFBA, PFOA, and PFOS. Dashed lines represent MDLs. Trend analysis completed for subsets of PFBA, PFOA, and PFOS, with doubling time (*t*_2_) and half-lives (*t*_1/2_) stated.

No clear trends are apparent for TFA and PFPrA in Arctic air over the 2014–2023 period, reflected in long doubling times along with low correlation between modeled trends and measured data (*R* ^2^ < 0.6, Table S24). Trend analysis for HF2OH and HFIPA was not performed due to the semiquantitative analytical figures of merit for these compounds. The absence of a temporal trend of TFA in remote atmospheric samples recorded here stands in contrast to the rapid increases observed in other environmental matrices.^14^ For example, TFA concentrations increased approximately 10-fold in San Francisco Bay-area surface waters between 1998 and 2021 ^58^ and 4-fold in poplar leaves collected in German national parks between 1989 and 2019.^7^ This discrepancy can be partly explained by atmospheric modeling studies of TFA production from HFO-1234yf degradation, which predict that tropospheric TFA concentration increases will be approximately 5-fold lower in Arctic regions compared to Central Europe and the Western Continental United States.^54^

### Sources and Seasonal Behavior of Short-Chain PFAS

Relationships between levels of frequently detected PFASs (DF > 50%) and temperature were investigated by plotting the natural logarithm of the air concentration against inverse temperature, averaged over the weeklong sampling interval (1/T (K), 2014–2023, Table S20) and by calculating Spearman correlations. Only data above the MDL were used in the correlation analysis. Air concentrations of HFIPA, HF2OH, and TFA were significantly (*p* < 0.01) correlated with 1/T (Figure S8), unlike those of PFPrA, PFBA, PFHxA, PFOS, PFOA, PFNA, and PFDA. This lack of correlation is consistent with past reports and suggests that these PFASs are advected into, or formed in, the Arctic atmosphere via temperature-independent mechanisms.^16^ The moderate negative Spearman correlations for TFA (*s* = −0.653, *p* < 0.0001) and HF2OH (*s* = −0.457, *p* < 0.0001) indicate higher concentrations during summer (e.g., HF2OH in Figure 3) and suggest the possibility of local evaporation from either snow or the ocean during summertime. Interestingly, HFIPA displays inverse seasonality (*s* = 0.717, *p* < 0.0001), with concentrations peaking in winter (Figure S9). Winter air concentration maxima may be attributed to two factors: (1) reduced breakthrough due to increased sorption capacity at lower temperatures and (2) decreased photodegradation during winter months. Similar seasonal patterns have been observed for hexachlorobutadiene.^59^

Recent modeling work has supported ionic PFAS to be present significantly in the gas-phase under environmentally relevant conditions, with scPFAS particle-binding dominated by association with aqueous aerosols.^30^ This gas-phase propensity may enable atmospheric redistribution following snowmelt. To investigate the snowmelt as a local source of atmospheric scPFAS, we identified melt events for 2014–2023 using albedo, soil temperature, and average air temperature. Samples were grouped into premelt, melt, and postmelt groups, as described in S9, and subjected to statistical analysis. Concentrations of HFIPA, HF2OH, and TFA were significantly different (*p* < 0.01) between groups according to Kruskal–Wallis ANOVA, with full results given in Tables S21 and S22. Dunn’s test was used to compare groups. A significant increase in TFA concentration between the premelt and melt periods (Figure 4) suggests the release of precursors or TFA itself from the snowpack to the atmosphere. Recently, the median deposition flux of TFA onto surface snow in Foxfonna, Svalbard, was found to be 36-times greater during summer compared to that during winter.^17^ With a lack of correlation between TFA snow concentrations and Na^+^, as well as recent reporting of scPFAS in high-altitude glaciers, it is proposed that precursor atmospheric degradation is the major source of scPFAS in the remote Arctic.^3,17,18^ Similar behavior with higher levels during melt was observed for HF2OH, which has precursor contributions from any chemical that can undergo environmental oxidation into HFA, such as hexafluoroisobutylene (HFIB), 1,1,1,3,3,3-hexafluoropropane (HFC-236fa), and hexafluoropropylene oxide (HFPO).^9,60–62^ In contrast, HFIPA was found to have significantly higher air concentrations before the melt event, with comparisons between melt and postmelt groups lacking statistical significance. Our field measurements therefore suggest that the Arctic snowmelt event is a contributing factor to the atmospheric concentrations of HF2OH and TFA or their precursors.

**Figure 4:**
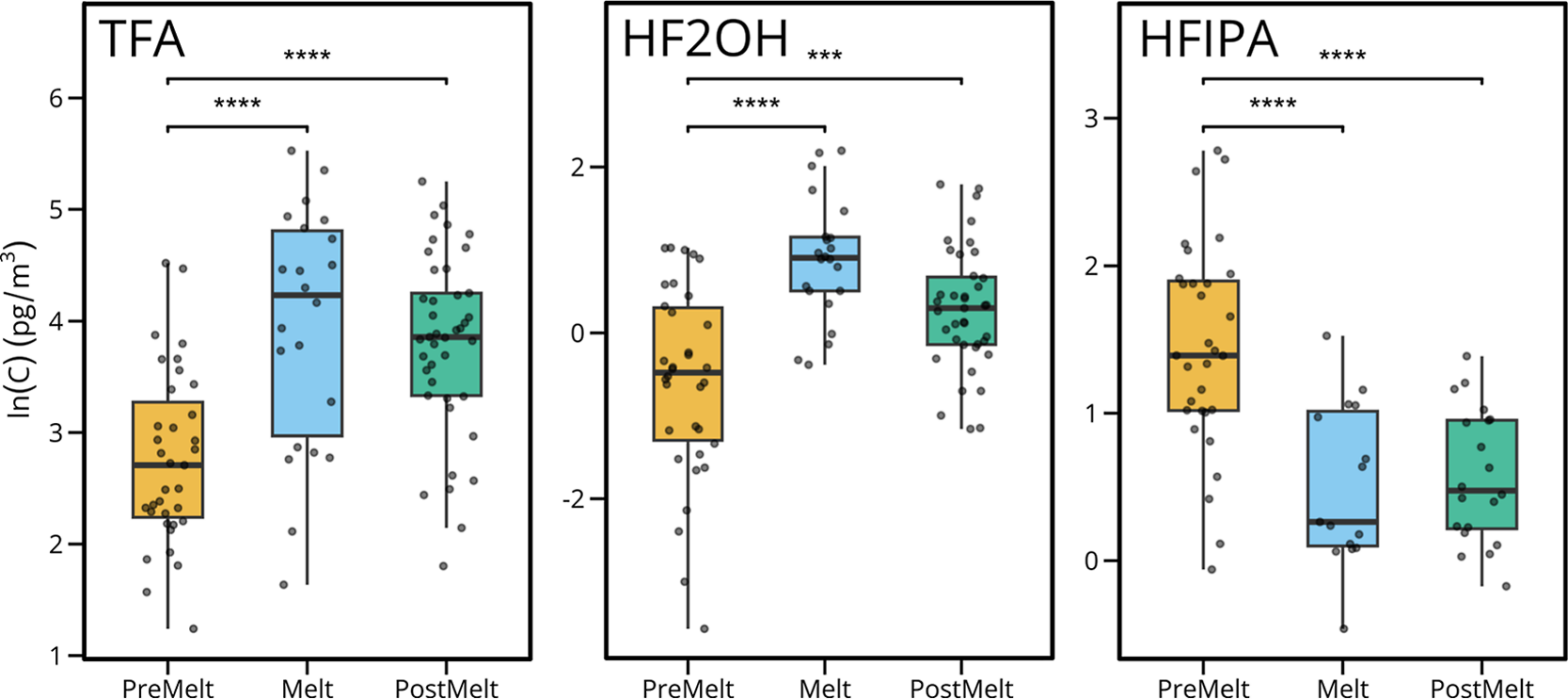
Pairwise comparisons of TFA, HF2OH, and HFIPA concentrations across melt stages. Kruskal–Wallis analysis followed by Dunn’s posthoc test revealed significant differences in TFA between Melt and PreMelt stages (*p* = 0.00001) and between PostMelt and PreMelt stages (*p* = 0.00002). HF2OH showed similar patterns, with significant differences between Melt and PreMelt stages and between PostMelt and PreMelt stages (both *p* = 0.00001). HFIPA displayed the reverse pattern, with significantly higher concentrations in the PreMelt period. Statistical significance: **p ≤ 0.01, ***p ≤ 0.001, ****p ≤ 0.0001.

Calculations estimating equilibrium partitioning of compounds between the different phases (gas phase, ice surface, organic particles, meltwater) within a melting Arctic snowpack at 0 °C (Figure S6C)^27^ indicate that such precursor chemicals would be expected to be predominantly in the air pore space of the snowpack, suggesting limited possibility for such chemicals to accumulate in snow. For HF2OH, significant sorption to the ice surface is predicted, suggesting potential accumulation in the snowpack (Figure S6A). The high uncertainty in the estimated log *K*_AW_ for HF2OH means that the relative preference for sorption to the ice surface or dissolution in meltwater is also uncertain. HFIPA is estimated to have an equal preference for these two phases (Figure S6B). The calculations suggest that during snowmelt, both HFIPA and HF2OH would be released with the meltwater and not to the atmosphere upon snowmelt. As such, these simple calculations fail to provide a mechanistic explanation for the divergent influence of snowmelt on the air concentrations of different scPFAS. This discrepancy highlights a challenge, as modeling techniques validated for nonpolar POPs may be less effective for neutral PFAS, as has been shown for basic physiochemical property prediction.^63^

### Implications

Our work expands on the understanding of scPFAS air concentrations to the remote Arctic, with concentration levels of TFA and PFPrA reported. The absence of long-term trends for these compounds over 2014–2023 contrasts with increasing concentrations of TFA in other environmental compartments globally. We note that scPFASs are expected to accumulate in the hydrosphere, and their transient existence in the remote atmosphere may not be able to capture increasing trends as effectively as ice-core records or archived plant media. Compared to the much higher air concentrations measured in urban locations, our data may represent the lower bound of global atmospheric scPFAS concentrations, which serve to define environmentally relevant concentrations to understand their health and environmental impacts.

We also present the discovery of HFIPA and HF2OH at high concentrations (>1 pg/m^3^) in Arctic air. Their demonstrated LRET, additionally supported by modeling, suggests their ubiquity in the atmosphere. This contrasts with their limited reporting in environmental compartments. Priority should be given to understanding their sources, concentrations within remote ecosystems, and the risks posed by the environmental presence of these rarely reported scPFASs. In addition, our work suggests the existence of complex cycling of TFA, HF2OH, and HFIPA air concentrations in the Arctic, with temperature, photochemical degradation, and local re-emission as contributing factors. Future work aimed at deconvoluting these factors via online or higher time resolution measurements will be imperative to understanding their behavior in the polar troposphere.

Finally, our updated long-term trends for PFBA, PFOA, and PFOS reflect increasing concentrations over the past 5 years. For PFOA and PFOS, the reversal follows their listing on the Stockholm Convention and correlates with reported shifts in emissions contributions from the United States and Europe to China. Identifying the industrial sources of these compounds and developing a framework toward their elimination is critical to maintaining the effectiveness of the Convention and to avoid them from joining the “POP purgatory” of listed compounds with significant unknown and unintentional sources, such as HCB and HCBD.

## Supplementary Material



## Notes

Detailed descriptions of the sample preparation and analysis employed, targeted chemicals of interest, instrumental methods and analytical figures of merit, suspect-screening details, evaluation of long-range transport potential, snow partitioning calculations, correlations between air concentrations and snowmelt events, and long-term trend analysis (PDF)
