# Supplementary material for: Extending the Monitoring of Perfluoroalkyl Substances in Arctic Air Reveals a High Abundance of Both Short Acids and Neutral Compounds
Source: Environ Sci Technol. 2025 Aug 1;59(31):16533–42. doi: 10.1021/acs.est.5c05145 (PMC12355948; doi:10.1021/acs.est.5c05145)
Supplement: Supplementary file 1 [file es5c05145_si_001.pdf]

Supporting Information

Extending the monitoring of perfluoroalkyl substances in Arctic air reveals a high abundance of both short acids and neutral compounds

*Alexander Kasperkiewicz[a,b], Frank Wania[b,d], Fiona Wong[a], Alexander Vlasenko[c], Henrik Li[c], Jared Chisamore[a], Helena Dryfhout-Clark[a], Phil Fellin[c], Hayley Hung[a, *]*

[a] Air Quality Processes Research Section, Environment and Climate Change Canada, 4905 Dufferin Street, Toronto, Ontario M3H 5T4, Canada
[b] Department of Chemical Engineering and Applied Chemistry, University of Toronto, Toronto, ON M5S 3E5, Canada
[c] Airzone One Ltd., 222 Matheson Blvd. E., Mississauga, Ontario L4Z 1X1, Canada
[d] Department of Physical and Environmental Sciences, University of Toronto Scarborough, Toronto, ON M1C 1A4, Canada

This supporting file includes: 30 pages, 24 tables and 9 figures

*Corresponding Author:
Hayley Hung
Air Quality Processes Research Section
Environment and Climate Change Canada
4905 Dufferin Street
Toronto, Ontario, M3H 5T4
Telephone: 1-416-739-5944
E-mail: hayley.hung@ec.gc.ca

Table of Contents

No.	Section	Page
S1	Sample preparation and analysis details	S3
S2	Chemicals of interest	S4
S3	Instrumental method details	S5
S4	Design of Experiment: Box-Behnken	S8
S5	Analytical figures of merit	S11
S6	Suspect-screening processing details	S18
S7	Evaluation of long-range transport potential	S20
S8	Snow partitioning calculations	S22
S9	Correlation of air concentrations to snowmelt events	S24
S10	Trend analysis details and results	S28
S11	References	S29

S1. Sample preparation and analysis details

The GFF and PUF/XAD/PUF sorbent cartridge were extracted separately for all the samples (n = 204), field blanks (n = 68), and lab blanks (n = 32) collected throughout the sampling period of March 3, 2014, to October 2, 2023. In Table S1, the parameters of the accelerated solvent extraction (ASE 200, Dionex Corporation, Sunnyvale, CA, USA) protocol used are shared. Prior to extraction, the samples were spiked with 50 µL of the internal standard (IS) panel at 0.04 ng·µL^{-1} containing $^{18}O_2$-PFHxS, $^{13}C_4$-PFOS, $^{13}C_4$-PFOA, $^{13}C_4$-PFBA, $^{13}C_5$-PFNA, $^{13}C_2$-PFHxA, $^{13}C_2$-PFDA¸$^{13}C_2$-PFUnDA¸$^{13}C_2$-PFDoDA¸ and $^{13}C_8$-FOSA, procured from Wellington Laboratories (Guelph, ON, Canada). The methanol fraction was concentrated by rotary evaporation followed by nitrogen blow-down to 0.5 mL. Due to the focus on ionic PFAS, only the methanol fraction was analyzed in this work. Prior to instrumental analysis, 10 µL of 0.01 ng·µL^{-1} of $^{13}C_8$-PFOS and $^{13}C_8$-PFOA (Wellington Laboratories, Guelph, ON, Canada) and 10 µL of 0.1 ng·µL^{-1} of $^{13}C_2$-TFA and $^{13}C_3$-PFPrA (Cambridge Isotope Laboratories, Inc., Andover, MA, USA) were added to the extract as injection standards (InjS).

Due to the long-term sample collection and analysis of the sample set, there are challenges in equivalent sample treatment over the full sample range. Samples collected from March 3, 2014 to October 8, 2018 (n = 99), along with relevant blanks, were analyzed for ionic PFAS with $n_C \geq 4$ and reported in previous work.[1] This analysis was performed using a method run using an Acquity I-Class Ultra-Performance Liquid Chromatography (UPLC) system coupled with Xevo TQ-S triple quadrupole mass spectrometer (MS/MS) (Waters Corporation, Milford, MA, USA) operated in MRM mode. Samples collected from November 5, 2018 to October 2, 2023 (n = 105) along with relevant blanks, were analyzed for the conserved target list from previous work along with the newly added short iPFAS enabled from the developed method in this work. The full sample set (n = 204) was analyzed using the developed method, however only short-chain compounds results were added to the dataset, and the iPFAS results previously reported were not reprocessed.

Quantification was completed using the isotope-dilution method, with internal standards paired with matching native compounds when possible and paired prioritizing chemical class and retention time when a corresponding isotopologue was not available. The analytical figures of merit for the quantified compounds can be found in Table S7. As $^{13}C_2$-TFA and $^{13}C_3$-PFPrA were added prior to analysis and not prior to sample extraction, the results must be treated as semi-quantitative due to a lack of correction for extraction efficiency, and possible sample loss. External recovery results are presented in Table S10. Similarly, PUF/XAD/PUF samples extracted in years 2020 and 2021 were externally corrected via added InjS $^{13}C_8$-PFOS and $^{13}C_8$-PFOA only, due to spiking error during the extraction process.

Table S1. Accelerated solvent extraction method details*

Parameter	Setpoint
Extraction temperature	50 °C

Pressure	800 PSI
Preheat time	1 min
Heat up time	5 min
Static cycle time	5 min
Flush volume	60 %
Cycles	2

*All samples are extracted into two fractions, the first fraction with hexane and the second with methanol. Only the second fraction was analyzed in this work.

S2. Chemicals of interest

Table S2. Abbreviations, names, chemical formula, and CAS numbers of chemicals of interest

Abbreviation	Name	Chemical Formula	CAS Number
TFA	Trifluoroacetic acid	$C_2HF_3O_2$	76-05-1
PFPrA	Perfluoropropanoic acid	$C_3HF_5O_2$	422-64-0
PFBA	Perfluorobutanoic acid	$C_4HF_7O_2$	375-22-4
PFPeA	Perfluoropentanoic acid	$C_5HF_9O_2$	2706-90-3
PFHxA	Perfluorohexanoic acid	$C_6HF_{11}O_2$	307-24-4
PFHpA	Perfluoroheptanoic acid	$C_7HF_{13}O_2$	375-85-9
PFOA	Perfluorooctanoic acid	$C_8HF_{15}O_2$	335-67-1
PFNA	Perfluorononanoic acid	$C_9HF_{17}O_2$	375-95-1
PFDA	Perfluorodecanoic acid	$C_{10}HF_{19}O_2$	335-76-2
PFUnDA	Perfluoroundecanoic acid	$C_{11}HF_{21}O_2$	2058-94-8
PFDoDA	Perfluorododecanoic acid	$C_{12}HF_{23}O_2$	307-55-1
PFTrDA	Perfluorotridecanoic acid	$C_{13}HF_{25}O_2$	72629-94-8
PFTeDA	Perfluorotetradecanoic acid	$C_{14}HF_{27}O_2$	376-06-7
PFHxDA	Perfluorohexadecanoic acid	$C_{16}HF_{31}O_2$	67905-19-5
PFODA	Perfluorooctadecanoic acid	$C_{18}HF_{35}O_2$	16517-11-6
PFBS	Perfluorobutanesulfonic acid	$C_4HF_9O_3S$	375-73-5
PFHxS	Perfluorohexanesulfonic acid	$C_6HF_{13}O_3S$	355-46-4
PFOS	Perfluorooctanesulfonic acid	$C_8HF_{17}O_3S$	1763-23-1
PFDS	Perfluorodecanesulfonic acid	$C_{10}HF_{21}O_3S$	335-77-3
HFPO-DA	2,3,3,3-Tetrafluoro-2-(1,1,2,2,3,3,3-heptafluoropropoxy)propanoic acid	$C_6HF_{11}O_3$	13252-13-6
FBSA	Perfluorobutanesulfonamide	$C_4H_2F_9NO_2S$	30334-69-1
FHxSA	Perfluorohexanesulfonamide	$C_6H_2F_{13}NO_2S$	41997-13-1
FHpSA	Perfluoroheptanesulfonamide	$C_7H_2F_{15}NO_2S$	68259-15-4
FOSA	Perfluorooctanesulfonamide	$C_8H_2F_{17}NO_2S$	754-91-6
FDSA	Perfluorodecanesulfonamide	$C_{10}H_2F_{21}NO_2S$	27619-90-5
PFECHS	Perfluoro-4-ethylcyclohexanesulfonic acid	$C_8HF_{15}O_3S$	646-83-3
HF2OH	1,1,1,3,3,3-Hexafluoropropane-2,2-diol	$C_3H_2F_6O_2$	920-66-1
HFIPA	1,1,1,3,3,3-Hexafluoropropan-2-ol	$C_3H_2F_6O$	920-66-1

S3. Instrumental method details

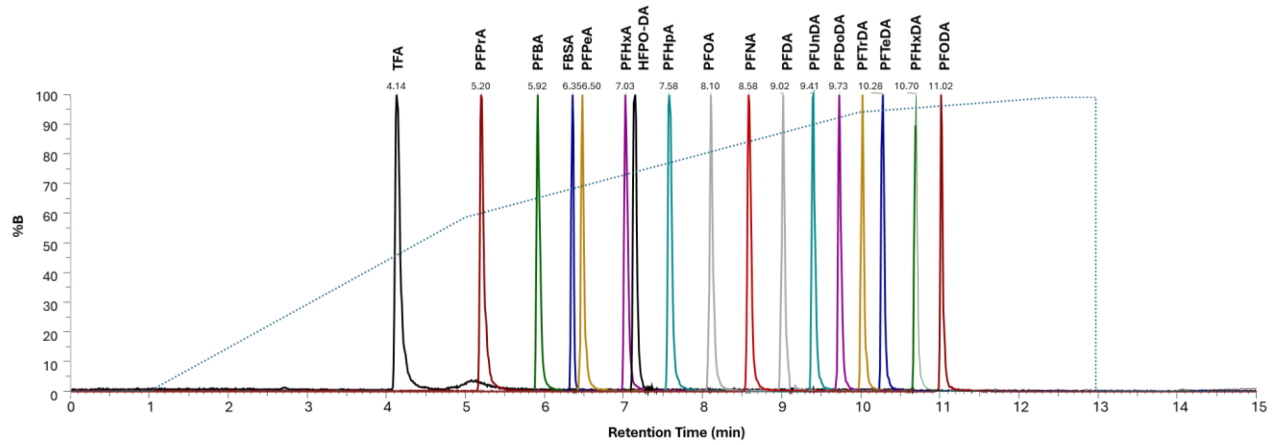

Figure S1. Chromatogram of a subset of the PFAS in the target list generated using the developed method. The gradient program is overlaid in %B terms.

Table S3. Liquid chromatographic separation method details

**Instrument**	ThermoFisher Vanquish Binary Pump (VH-P10-A)<br>ThermoFisher Vanquish Autosampler (VH-A10-A)<br>ThermoFisher Vanquish Column Compartment (VH-A10-A)	
**Delay Column**	Waters Isolator Column	
**Column**	Waters Atlantis Premier BEH C18 AX Column, 1.7 µm, 2.1 x 50 mm + Waters ACQUITY UPLC BEH C18 VanGuard Pre-column, 130Å, 1.7 µm, 2.1 mm X 5 mm	
**Column Temperature**	45 °C	
**Sample Temperature**	15 °C	
**Mobile Phase A**	Water + 0.025 % Acetic Acid	
**Mobile Phase B**	Methanol + 20 mM Ammonium Acetate	
**Flow Rate**	0.4 mL/min	
**Gradient Program**	**Time (min)**	**%B**
	0.000	2.0
	1.000	2.0
	5.000	60.0
	10.000	95.0
	12.500	100.0
	13.000	100.0
	13.001	2.0
	15.000	2.0
**Injection Volume**	4 µL	
**Length of Method**	16 min	
**Initial Condition Backpressure**	5800 PSI	

Table S4. Mass spectrometry method details

**Instrument**	Orbitrap Exploris 240	
**Application Mode**	Small Molecule	
**Method Duration**	15 min	
**Ion Source Parameters**	Ion Source Type	H-ESI
	Spray Voltage	Static
	Negative Ion (V)	3000
	Sheath Gas	50
	Aux Gas	10
	Sweep Gas	1
	Ion Transfer Tube Temp (°C)	200
	Vaporizer Temp (°C)	325
**Master Scan Parameters**	Resolution	90000
	Scan Range (m/z)	80-1000
	RF Lens (%)	70
	AGC Target	Standard
	µScans	1
	Data Type	Profile
	Polarity	Negative
	Source Fragmentation	Disabled
**Filter Tree**	MS → Targeted Mass → Dynamic Exclusion → ddMS^2	
**Targeted Mass List**	Time Mode: Retention Time Window, m/z	
**Dynamic Exclusion Parameters**	Exclude after 1 time	
	Exclusion duration of 3 s	
	Mass Tolerance of 5 ppm	
**ddMS^2 Parameters**	Multiplex Ions	False
	Isolation Window (m/z)	2
	Collision Energy Type	Normalized
	HCD Collision Energies (%)	15, 55
	Orbitrap Resolution	15000
	First Mass (m/z)	60
	AGC Target	Standard
	Maximum Injection Time	Auto
	µScans	1
	Data Type	Centroid
**DIA Parameters**	Multiplex Ions	False
	Isolation Window (m/z)	2
	Collision Energy Type	Normalized
	HCD Collision Energies (%)	15, 55
	Orbitrap Resolution	15000
	DIA Mass Table Ranges (m/z)	90-150
		150-200
		200-250
		250-350
		350-450
		450-650
		650-1000
	AGC Target	Standard
	Maximum Injection Time	Auto
	µScans	1
	Data Type	Centroid

Table S5. Targeted mass list for ddMS^2

Compound	m/z	Time (min)
TFA	112.9856	4.1
PFPrA	162.9824	5.1
PFBA	212.9792	5.9
PFPrA	262.976	6.4
PFHxA	312.9728	7
PFHpA	362.9696	7.5
PFOA	412.9664	8.1
PFNA	462.9632	8.6
PFDA	512.96	9
PFUnDA	562.9568	9.4
PFDoDA	612.9537	9.7
PFTrDA	662.9505	10
PFTeDA	712.9473	10.3
PFHxDA	812.9409	10.7
PFODA	912.9345	11
PFBS	298.943	6.3
PFHxS	398.9366	7.4
PFOS	498.9302	8.3
PFDS	598.9238	9.1
FBSA	297.959	6.4
FHxSA	397.9526	7.5
FHpSA	447.9494	8.1
FOSA	497.9462	8.5
FDSA	597.9398	9.4
HFPO-DA-CO2H	284.9779	7.1
PFECHS	460.9334	7.81

S4. Design of Experiment: Box-Behnken

Perfluorocarboxylic acids (PFCA) are susceptible to in-source fragmentation via electrospray ionization due to the labile carboxylic acid moiety. Excessive in-source fragmentation can increase instrument detection limits and reduce confidence in analysis. Ion source parameters were varied in search of a set of conditions which would minimize in-source fragmentation of PFCAs without compromising sensitivity for other chemical families such as perfluorosulfonic acids and perflurosulfonamides. Specifically, we used a Box-Behnken response surface (Table S6) to explore the impact of ion transfer tube temperature (200 – 350 °C), vaporizer temperature (250 – 400 °C), and voltage applied to the vaporizer (-2000 – -3500 V) on the observed in-source fragmentation. The design was created and analysed using the Design of Experiments (OriginLab Technical Support, v1.42) application within OriginPro 2024b (OriginLab Corporation, Northampton, USA). Data was collected using the same instrumental parameters as in S3. The response (*i.e.* observed peak areas normalized to the maximum and expressed as a ratio) was analyzed for each target using a full quadratic model, at a 95 % confidence level, with selected contour plots given in Figs. S2 and S3.

Across the parameter range tested, two general behaviours were observed with respect to normalized area of the parent molecules. For PFCAs and HFPO-DA, the main significant effect was found to be the transfer line temperature, with operation at 200 °C allowing for 3 to 10-fold gains in normalized areas observed when compared to 350 °C. The vaporization temperature was on the threshold of significance ($p >= 0.05$) for this compound group, and thus contour plots are shown at a constant vaporization temperature of 325 °C in Fig. S2.

Additionally, gentle ionization conditions also yielded the highest normalized areas for HFPO-DA. HFPO-DA and related compounds are notably thermally liable which challenges their detection with MS instrumentation. Even at the mildest ESI conditions tested, the parent molecule was observed at significantly lower intensities than the in-source fragment, with further fragmentation observed yielding the $[C_3F_7]^-$ fragment at 168.9894 m/z. The response surface for in-source fragment $[M – CO_2H]^-$ for HFPO-DA is shown in Fig. S3. Conversely, response surface for parent molecules of the sulfonic acid and sulfonamide class was observed to be less variable. The transfer line temperature was not found to be a significant effect for all compounds of these classes, with the vaporization temperature impacting normalized areas predominantly. The response surface of PFHxS is shown below in Figure S3 with the transfer temperature held constant at 200 °C. Following the analysis of the DoE data, the ion source parameters were selected prioritizing the normalized areas of compounds prone to in-source fragmentation. The selected ionization source parameters were a transfer line temperature of 200 °C, a vaporization temperature of 325 °C, and an electrospray voltage of -3000 V.

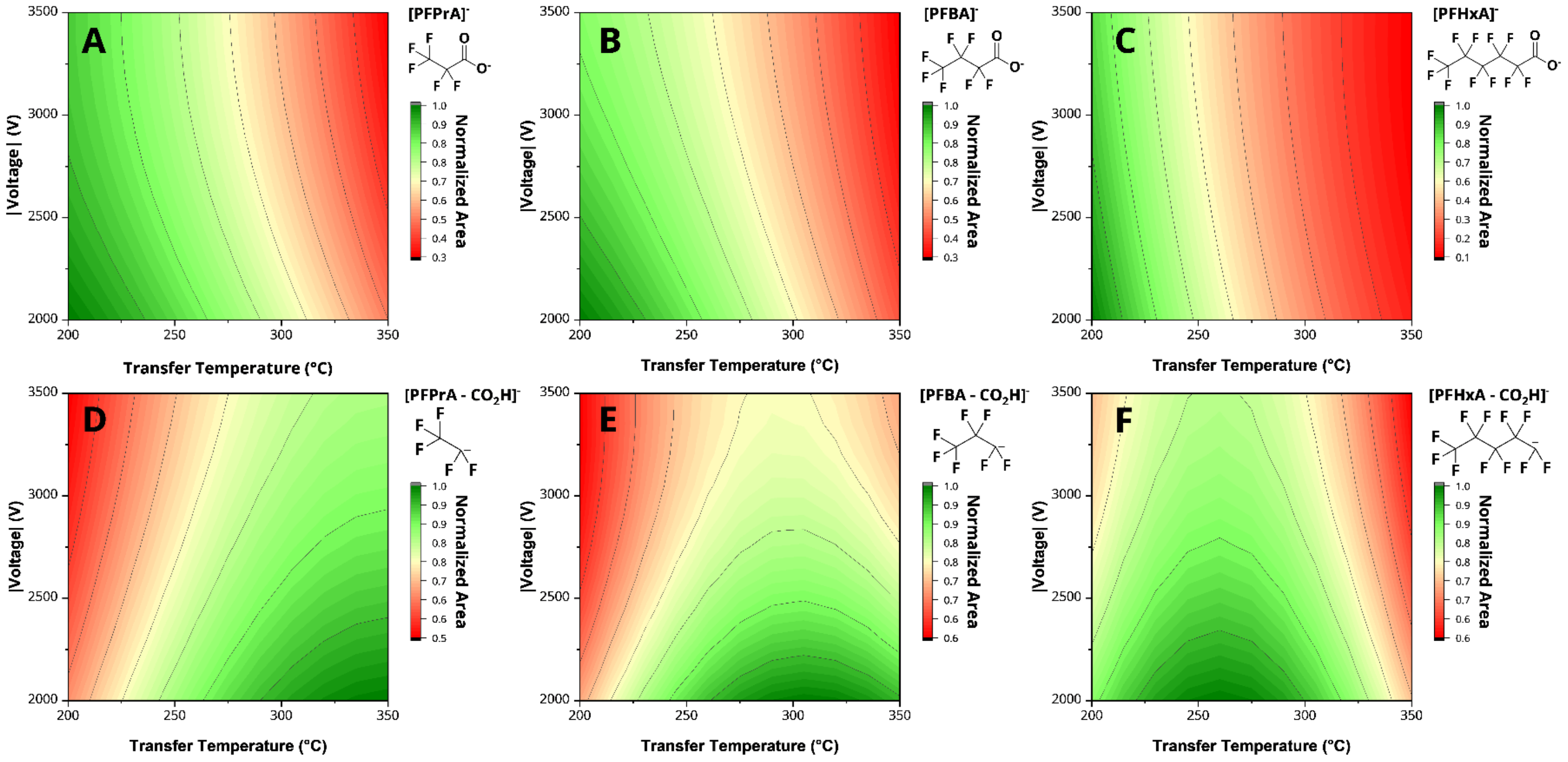

Figure S2. Surface response plots generated using normalized peak areas measured for PFPrA (A), PFBA (B), and PFHxA (C) along with their corresponding in-source fragments (D, E, F) due to the loss of the carboxylic acid moiety. The response surface and significant effect of transfer temperature was similar for all PFCAs monitored, with gentler ionization conditions (lower transfer temperature and lower ionization voltage) resulting in up to 10-fold gains across normalized areas within the conditions tested. Generally, the peak area variability of the in-source fragment was observed to be less drastic across the surface, with peak relative areas observed at higher transfer temperatures. In the cases of both PFCAs and their in-source fragments, the transfer temperature and ionization voltages were found to impact normalized areas more than the vaporization temperature across the response surface.

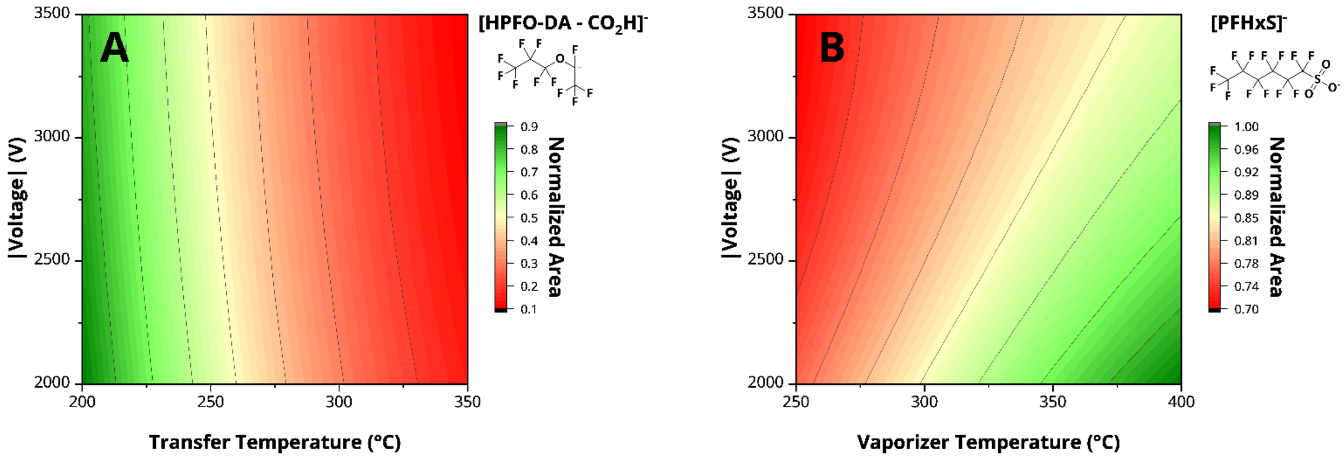

Figure S3. Surface response plots generated using normalized peak areas measured for the in-source fragment of HPFO-DA (A) and PFHxS (B). Relative area changes of greater than 10-fold were observed over the conditions tested for HPFO-DA. Due to the carboxylic acid moiety and ether linkage, this compound was the most susceptible in the panel to in-source fragmentation. Similar ranking of significant effects was observed to the PFCA-class of compounds, with transfer line temperature impacting signal observed most strongly. The response surface across the perfluorosulfonic acid and perfluorosulfonamide groups of chemicals was observed to be less variable (as shown for PFHxS in B), with the transfer temperature exerting a non-significant effect on normalized areas. In B, the transfer temperature was kept constant at 200 °C to generate the contour plot.

Table S6. Box-Behnken experimental parameters used in the optimization of ion source conditions

Std Order	Voltage (V)	Transfer Temp (°C)	Vaporizer Temp (°C)
1	-2000	200	325
2	-3500	200	325
9	-2750	200	250
11	-2750	200	400
5	-2000	275	250
6	-3500	275	250
7	-2000	275	400
8	-3500	275	400
13	-2750	275	325
14	-2750	275	325
15	-2750	275	325
16	-2750	275	325
3	-2000	350	325
4	-3500	350	325
10	-2750	350	250
12	-2750	350	400

S5. Analytical figures of merit

Estimation of Instrumental Limit of Detection (LOD)

A signal-to-noise approach for LOD calculation can produce unrealistic results for HRMS instrumentation due low or undetectable noise. Thus, the LOD for each target was calculated based on the standard deviation of the residuals ($S_{y,x}$) of the calibration points, across the lowest 5-level range of the calibration curve, with the lowest level on the threshold of visual LOD. Conservatively, the LOD was reported as the closest calibration level value above the calculated LOD and was visually verified. LODs are reported in both sample terms ($pg \cdot m^{-3}$) and in absolute terms ($pg\ on\ column$). All LOD values for quantified compounds are reported in Table S7.

$$(1)\ LOD\ (pg \cdot m^{-3}) = 3.3 \cdot \frac{S_{y,x}}{slope}$$

Estimation of Method Detection Limit (MDL)

Due to the batch-extraction nature of the long-term air monitoring sample set, MDLs were calculated on an extraction batch basis. They are defined as the mean plus three times the standard deviation of the blanks (both field and laboratory blanks) corresponding to an extraction batch or year. For blanks where no signal was detected, a substitution of 2/3 of the LOD for the compound was made. For compounds in which no signal was detected across all blanks, 3 × LOD was used as the MDL. Values below MDL were removed from trend analysis, and MDLs are displayed on trend analysis plots as dashed lines.

Table S7. Analytical figures of merit for the compound panel following method development. LODs have been noted to vary from batch to batch (year to year) by 1-2 calibration levels. CC: Calibration Curve.

Compound	RT (min)	Internal Standard	Prepared Calibration Range (pg)		Slope	Intercept	R^2	LOD (pg in CC Level)	LOD (pg/m^3)	LOD (pg on column)
TFA	4.1	$^{13}C_2$-TFA	125	250000	9.6E-04	1.1E-01	0.9988	500	0.25	4
PFPrA	5.12	$^{13}C_3$-PFPrA	2.5	125000	8.8E-04	4.6E-02	0.9996	250	0.125	2
PFBA	5.9	$^{13}C_4$-PFBA	2.5	125000	4.2E-04	9.5E-04	0.9996	50	0.025	0.4
PFPeA	6.45	$^{13}C_4$-PFBA	2.5	5000	8.7E-03	1.9E-02	0.9977	50	0.025	0.4
PFHxA	7	$^{13}C_2$-PFHxA	2.5	5000	1.1E-02	1.8E-02	0.9963	25	0.0125	0.2
PFHpA	7.54	$^{13}C_2$-PFHxA	2.5	5000	1.4E-02	5.0E-02	0.9975	25	0.0125	0.2
PFOA	7.81	$^{13}C_4$-PFOA	2.5	5000	9.3E-04	3.9E-03	0.9991	25	0.0125	0.2
PFNA	8.55	$^{13}C_5$-PFNA	2.5	5000	4.8E-04	5.1E-05	0.9994	25	0.0125	0.2
PFDA	8.99	$^{13}C_2$-PFDA	2.5	5000	4.8E-04	-3.2E-04	0.9993	25	0.0125	0.2
PFUnDA	9.36	$^{13}C_2$-PFUnDA	2.5	5000	5.1E-04	1.1E-03	0.9994	50	0.025	0.4
PFDoDA	9.7	$^{13}C_2$-PFDoDA	2.5	5000	4.8E-04	-8.8E-04	0.9993	50	0.025	0.4
PFTrDA	9.99	$^{13}C_2$-PFDoDA	2.5	5000	4.1E-04	-4.8E-04	0.9969	50	0.025	0.4
PFTeDA	10.25	$^{13}C_2$-PFDoDA	2.5	5000	3.4E-04	-2.1E-03	0.9969	50	0.025	0.4
PFHxDA	10.67	$^{13}C_2$-PFDoDA	2.5	5000	2.5E-04	-2.1E-03	0.9976	125	0.0625	1
PFODA	11	$^{13}C_2$-PFDoDA	2.5	5000	1.8E-04	-3.3E-03	0.9939	125	0.0625	1
PFBS	6.36	$^{18}O_2$-PFHxS	2.5	5000	6.1E-04	3.4E-04	0.9992	6.66	0.00333	0.05
PFHxS	7.37	$^{18}O_2$-PFHxS	2.5	5000	5.7E-04	4.0E-03	0.9994	6.66	0.00333	0.05
PFOS	8.33	$^{13}C_4$-PFOS	2.5	5000	5.0E-04	2.4E-04	0.9996	6.66	0.00333	0.05
PFDS	9.13	$^{13}C_4$-PFOS	2.5	5000	4.5E-04	-8.8E-05	0.9987	25	0.0125	0.2
HFPO-DA	7.1	$^{13}C_2$-PFHxA	2.5	5000	1.9E-03	-5.0E-03	0.9976	125	0.0625	1
FBSA	6.35	$^{13}C_8$-FOSA	2.5	5000	4.5E-04	3.1E-04	0.9989	6.66	0.00333	0.05
FHxSA	7.54	$^{13}C_8$-FOSA	2.5	5000	4.0E-04	1.2E-04	0.9987	6.66	0.00333	0.05
FHpSA	8.07	$^{13}C_8$-FOSA	2.5	5000	3.3E-04	1.6E-04	0.9981	6.66	0.00333	0.05
FOSA	8.57	$^{13}C_8$-FOSA	2.5	5000	5.2E-04	2.7E-04	0.9993	6.66	0.00333	0.05
FDSA	9.36	$^{13}C_8$-FOSA	2.5	5000	4.2E-04	4.8E-05	0.9972	6.66	0.00333	0.05
PFECHS	7.8	$^{13}C_4$-PFOS	2.5	5000	5.5E-04	-2.5E-04	0.9991	6.66	0.00333	0.05
HF2OH	4.27	$^{13}C_2$-TFA	2.5	250000	5.5E-04	-2.5E-04	0.9899	50	0.025	0.4
HFIPA	4.80	$^{13}C_3$-PFPrA	2.5	500000	9.1E-05	5.1E-02	0.9909	1000	0.5	8

Table S8. Comparison of detection limits reported in literature for scPFAS approaches. Values in fg are on-column absolute masses, values reported in pg/m^3 are per air sample volume, values reported in pg/µL are concentrations in sample vials prior to injection.

Compound	This Work (pg/m^3)	Wong et al. 2018 (pg/m^3)	This Work (fg)	Ye et al. 2023[2] (fg)	Björnsdotter et al. 2021[3] (fg)[b]	Hartz et al. 2024[4] (fg)[c]	Neuwald et al. 2022[5] (fg)[d]
TFA	0.25		4000 (1 pg/µL)[a]	55	400.4		0.95 pg/µL[a]
PFPrA	0.125		2000	156	400.4		3333.3
PFBA	0.025	0.0063	400	9	400.4		1250
PFPeA	0.025	0.013	400	15		506	125
PFHxA	0.0125	0.031	200	10		506	62.5
PFHpA	0.0125	0.013	200	8		506	250
PFOA	0.0125	0.0063	200	14		506	62.5
PFNA	0.0125	0.013	200	10		506	125
PFDA	0.0125	0.0063	200	17		506	62.5
PFUnDA	0.025	0.0063	400	18		506	62.5
PFDoDA	0.025	0.0063	400	14		506	62.5
PFTrDA	0.025	0.013	400			506	62.5
PFTeDA	0.025	0.0063	400	220		506	25
PFHxDA	0.0625	0.0063	1000			506	
PFODA	0.0625	0.013	1000			506	
PFBS	0.00333	0.0063	53.28			506	125
PFHxS	0.00333	0.0063	53.28			2002	125
PFOS	0.00333	0.0063	53.28			2002	125
PFDS	0.0125	0.0063	200			2002	62.5
HFPO-DA	0.0625		1000			506	250
FBSA	0.00333		53.28			506	
FHxSA	0.00333		53.28			506	
FHpSA	0.00333		53.28			506	
FOSA	0.00333		53.28			506	
FDSA	0.00333		53.28				
PFECHS	0.00333		53.28			506	

[a]DL was calculated based on concentration of target inside headspace vial prior to analysis

[b]DL in fg on column terms was calculated based on a sample volume of 2200 mL, a 100 µL pre-injection volume, and a 2 µL injection volume

[c]DL in fg on column terms was calculated based on a sample volume of 2200 mL, a 40 µL pre-injection volume, and a 4 µL injection volume

[d]DL in fg on column terms was calculated based on a sample volume of 200 mL, a 500 µL pre-injection volume, and a 7.5 µL injection volume for all analytes except PFPrA and TFA. For PFPrA, calculations based on a sample volume of 200 mL, a 1000 µL pre-injection volume, and a 5 µL injection volume were used. For TFA, concentration in the pre-injection vial was made using a sample volume of 19 mL, and a 4 mL pre-injection volume.

Table S9. Comparison of breakthrough % to the bottom PUF in the PUF/XAD/PUF sorbent pack. Normalization was completed as mass of target per gram of sorbent, using the mass of the bottom PUF and the mass of the top PUF + the XAD fraction. ND: Not detected.

	Absolute[1]		Normalized[2]	
Compound	Winter	Summer	Winter	Summer
TFA	5.5%	3.6%	9.2%	6.1%
PFPrA	8.5%	4.5%	14.0%	7.6%
HF2OH	4.5%	20.3%	7.6%	30.8%
HFIPA	ND	ND	ND	ND
PFBA	2.7%	8.8%	4.7%	14.5%
PFHxA	5.8%	13.2%	9.8%	21.1%
PFOA	2.9%	9.2%	4.9%	15.0%

[1]% mass on bottom PUF vs total
[2]% mass/g of sorbent on bottom PUF vs total

Table S10. Comparison of accuracy and precision (%RSD) of extracted spiked GFFs and PUF/XAD/PUF sorbent packs using the 2 fraction ASE extraction method used for all samples. Samples were spiked with 100 ng of TFA, 40 ng of PFPrA, HFIPA, HF2OH, and 20 ng of remaining compounds.

	Filter (n = 4)		PUF/XAD/PUF (n = 3)	
Compound	Accuracy (%)	Precision (%)	Accuracy (%)	Precision (%)
PFBA	98.6	4.6	79.4	6.8
FBSA			79.5	4.5
FDSA			87.5	3.6
FHpSA			84.3	6.1
FHxSA			86.3	6.8
FOSA			85.3	3.7
HFPO-DA	126.8	9.3	107.3	6.4
PFBS	96.1	3.8	83.0	8.7
PFDA	90.4	4.5	109.9	2.8
PFDoA	87.9	5.5	120.0	3.5
PFDS	94.8	4.1	102.5	6.4
PFECHS	105.9	4.5	98.4	4.9
PFHpA	101.0	4.5	103.7	5.0
PFHxA	104.8	4.1	106.2	5.7
PFHxDA	83.0	4.5	112.8	7.8
PFHxS	93.1	3.9	82.1	5.6
PFNA	95.8	4.0	102.7	2.1
PFOA	96.5	5.2	102.5	3.4
PFODA	82.7	6.1	129.1	9.4
PFOS	91.2	6.2	97.6	6.0
PFPeA	107.1	4.1	104.9	7.2
PFTeDA	84.9	5.2	108.8	3.3
PFTrDA	84.9	4.2	109.8	3.4
PFUdA	87.1	4.9	108.5	2.6
TFA	109.5	5.2	113.5	2.3
PFPrA	102.3	6.1	105.8	3.4
HF2OH			60.3	5.8
HFIPA			32.3	4.4

Table S11. Average recoveries of isotopically labelled internal standards for the sampling years in this work.

Sampling Year	PFBA - $^{13}C_4$	PFHxA- $^{13}C_2$	PFHxS- $^{18}O_2$	PFOA- $^{13}C_4$	PFNA- $^{13}C_5$	PFOS - $^{13}C_4$	PFDA- $^{13}C_2$	PFUnDA - $^{13}C_2$	PFDoDA - $^{13}C2$
2015	92%	87%	85%	86%	90%	85%	92%	93%	96%
2016	67%	61%	60%	60%	62%	60%	61%	62%	63%
2017	62%	61%	61%	62%	64%	61%	63%	68%	69%
2018	78%	67%	63%	64%	67%	64%	72%	68%	80%
2019	37%	43%	45%	49%	56%	56%	53%	56%	55%
2020	41%	41%	43%	45%	48%	45%	44%	45%	43%
2021	55%	53%	54%	53%	54%	55%	54%	54%	53%
2022	55%	54%	56%	55%	59%	57%	59%	61%	60%
2023	56%	51%	51%	57%	57%	57%	56%	55%	58%
All	59%	57%	56%	58%	61%	59%	61%	62%	63%
Standard Deviation, All	23%	19%	21%	18%	20%	20%	20%	21%	24%

Table S12. Method detection limits (pg/m^3) in this work for all compounds across all sampling years. For shaded cells, no detectable signal was found in blanks, and MDLs were calculated as 3 × LODs determined on a batch-to-batch basis.

**Compound**	**2014**	**2015**	**2016**	**2017**	**2018**	**2019**	**2020**	**2021**	**2022**	**2023**
TFA	4.86	6.97	5.90	5.76	7.84	2.26	1.56	1.33	2.68	3.13
PFPrA	0.31	0.22	0.14	0.42	0.51	0.13	0.05	0.09	0.11	0.10
PFBA	0.35	2.14	0.20	0.07	0.04	0.04	0.02	0.03	0.05	0.08
PFPeA	0.02	0.08	0.18	0.03	0.06	0.02	0.02	0.01	0.08	0.13
PFHxA	0.03	0.09	0.17	0.04	0.06	0.28	0.16	0.17	0.13	0.10
PFHpA	0.03	0.10	0.20	0.22	0.04	0.36	0.22	0.39	0.83	0.59
PFOA	0.12	0.17	0.19	0.14	0.12	0.16	0.18	0.10	0.09	0.18
PFNA	0.01	0.09	0.18	0.02	0.02	0.02	0.01	0.01	0.12	0.11
PFDA	0.03	0.10	0.17	0.04	0.01	0.10	0.06	0.07	0.03	0.03
PFUnDA	0.01	0.10	0.14	0.01	0.01	0.02	0.02	0.01	0.12	0.13
PFDoDA	0.01	0.09	0.16	0.01	0.01	0.02	0.02	0.01	0.10	0.12
PFTrDA	0.02	0.02	0.01	0.02	0.02	0.02	0.02	0.09	0.13	0.12
PFTeDA	0.01	0.09	0.15	0.01	0.01	0.02	0.02	0.08	0.09	0.11
PFHxDA	0.01	0.01	0.01	0.01	0.01	0.09	0.09	0.18	0.11	0.12
PFODA	0.04	0.01	0.02	0.02	0.02	0.08	0.09	0.17	0.10	0.10
PFBS	0.02	0.13	0.15	0.09	0.12	0.03	0.01	0.02	0.03	0.02
PFHxS	0.07	0.15	0.16	0.01	0.01	0.02	0.01	0.01	0.10	0.10
PFOS	0.04	0.08	0.10	0.01	0.01	0.02	0.03	0.01	0.10	0.11
PFDS	0.01	0.10	0.13	0.01	0.01	0.02	0.02	0.01	0.14	0.10
HFPO-DA										
FBSA										
FHxSA										
FHpSA										
FOSA	0.02	0.01	0.01	0.01	0.01				0.04	0.11
FDSA										
PFECHS										
HF2OH	0.02	0.16	2.65	0.02	0.04	0.08	0.07	0.17	0.06	0.05
HFIPA	0.97	0.92	4.24	0.76	0.85	1.51	0.76	0.54	0.27	0.25

Table S13. Descriptive statistics for high % DF compounds with data reported in pg/m^3, with n representing samples above MDL, over the March 2014 to October 2023 sampling interval. The mean is the arithmetic mean.

Compound	Maximum	Q3	Median	Mean	Q1	Minimum	DF %	n
TFA	251	47.6	19.6	37.3	9.84	1.76	80%	164
HFIPA	93.7	8.91	4.51	7.39	2.54	0.63	77%	153
PFBA	21.4	5.79	3.71	4.31	2.06	0.06	93%	190
HF2OH	9.00	1.80	0.79	1.32	0.32	0.03	87%	173
PFPrA	10.8	1.66	1.09	1.31	0.62	0.13	88%	180
PFHpA	3.17	1.00	0.66	0.73	0.25	0.06	44%	89
PFOA	1.28	0.43	0.32	0.35	0.23	0.11	65%	133
PFHxA	0.71	0.40	0.27	0.29	0.18	0.03	48%	97
PFNA	0.78	0.19	0.12	0.14	0.05	0.01	54%	111
PFDA	0.49	0.16	0.08	0.13	0.06	0.02	52%	106
PFBS	0.90	0.20	0.05	0.13	0.03	0.01	35%	72
PFOS	0.46	0.12	0.08	0.09	0.04	0.01	51%	105

Table S14. Descriptive statistics for low % DF compounds with data reported in pg/m^3, with n representing samples above MDL, over the March 2014 to October 2023 sampling interval. ND is not detected above the LOD. The mean is the arithmetic mean.

Compound	Maximum	Q3	Median	Mean	Q1	Minimum	DF %	n
PFPeA	0.35	0.10	0.06	0.07	0.04	0.01	30%	61
PFUnDA	0.19	0.04	0.02	0.04	0.02	0.01	23%	46
PFDoDA	0.09	0.03	0.02	0.02	0.01	0.01	16%	33
PFTrDA	0.08	0.03	0.02	0.03	0.02	0.01	6%	12
PFTeDA	0.17	0.04	0.04	0.05	0.03	0.02	3%	7
PFHxDA	0.01	0.01	0.01	0.01	0.01	0.01	1%	2
PFODA	0.06	0.05	0.05	0.05	0.04	0.04	3%	6
PFHxS	0.19	0.02	0.01	0.02	0.01	0.01	11%	23
PFDS	0.15	0.05	0.01	0.05	0.01	0.01	2%	4
FOSA	0.02	0.02	0.02	0.02	0.02	0.02	1%	2
FBSA				ND				
FHxSA				ND				
FHpSA				ND				
FDSA				ND				
HFPO-DA				ND				
PFECHS				ND				

S6. Suspect-screening processing details

Table S15. Workflow settings used in Compound Discoverer 3.3 SP3

Node	Details
**Workflow Description**	Find and identify Per- and Polyfluoroalkyl Substances (PFAS) through an untargeted workflow including retention time alignment, unknown compound detection, and grouping across all samples. Utilizes mzCloud (ddMS2), ChemSpider, and predicted mass spectral library for compound identification. Flags unknown compounds sharing fragment sets. Generates molecular networks.
**Discoverer Version**	3.3.3.200
**Select Spectra**	Spectrum Properties Filter (RT limit: 0), Scan Event Filters (Mass Analyzer: not specified, MS Order: Any, Collision Energy: 0-1000), Peak Filters (S/N Threshold: 1.5).
**Align Retention Times**	Alignment Model: Adaptive curve, Maximum Shift: 2 min, Mass Tolerance: 5 ppm, Remove Outlier: True
**Detect Compounds**	Mass Tolerance: 5 ppm, Minimum Peak Intensity: 1000, Trace Detection (Gaps to Correct: 2, Adjacent Non-Zeros: 2), Peak Detection (Chromatographic S/N Threshold: 1.5), Ions (various).
**Group Compounds**	Mass Tolerance: 5 ppm, RT Tolerance: 0.2 min, Align Peaks: False, Peak Rating Filter Threshold: 5
**Fill Gaps**	Mass Tolerance: 5 ppm, S/N Threshold: 1.5, Real Peak Detection: True, Restrictive Gap Filling: True
**Mark Background**	Max Sample/Blank: 5, Max Blank/Sample: 0, Hide Background: True

**Calculate Mass Defect**	Kendrick Mass Defect: True, Mass Defect Formulas: C F2
**Compound Class Scoring**	Compound Classes: PFAS General, S/N Threshold: 1, Mass Tolerance: 5 ppm
**Assign Compound Annotations**	Mass Tolerance: 5 ppm, Data Sources: mzCloud, ChemSpider, MassList, Predicted Compositions, Scoring Rules (Use mzLogic: True)
**Generate Molecular Networks**	Spectral Similarity (Full MSn Tree: True, Mass Shift: True), Transformations (e.g., Dehydration, Oxidation), Applied Filters (Min. MSn Score: 50, MSn Coverage: 70%)
**Search mzCloud**	Compound Classes: All, Precursor Mass Tolerance: 10 ppm, Fragment Mass Tolerance: 10 ppm, Max Results: 10
**Search ChemSpider**	Database: EPA DSSTox, Search Mode: By Formula or Mass, Mass Tolerance: 5 ppm, Max Results: 100
**Search Mass Lists**	Mass Lists: PFASSTRUCT-2022-04-20, PFAS_NEG, PFAS_NIST, RT Tolerance: 2 min, Mass Tolerance: 5 ppm
**Search mzVault**	Library: PFAS_CFM_specLibrary_Duke.db, Max Results: 10, Match Factor Threshold: 20, Mass Tolerance: 10 ppm
**Predict Compositions**	Mass Tolerance: 5 ppm, Element Counts (Min C F; Max C90 H190 Br3 Cl4 F75 N10 O18 P3 S5), Pattern Matching (Min Spectral Fit: 30%)

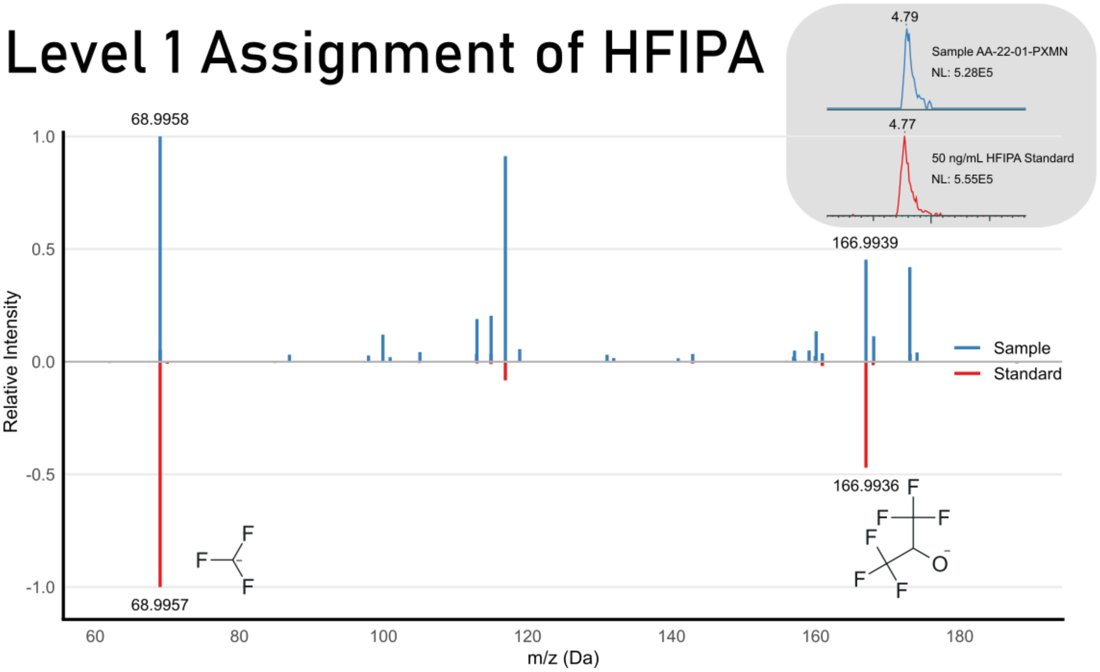

Figure S4. Confirmation of HFIPA in samples upon comparison to an HFIPA standard.

S7. Evaluation of long-range transport potential

Long-range transport potential (LRTP) was calculated using the modified LRTP Screening Tool (v0.99, 21.06.2022) described in Breivik et al.[6] Default environmental input parameters and activation energies (from EAS-E Suite) were used. The chemical parameters along with their sources are shown in Table S16. Figure S5 displays the results of the LRTP evaluation for HFIPA and HF2OH.

Table S16. Chemical parameters used in the emissions fraction approach Level III fugacity model (v0.99).[6]

Chemical	Formula Weight (g/mol)	$logK_{aw}$	$logK_{ow}$	k_{OH} at 25 °C ($\cdot 10^{-13}$ $cm^3 \cdot mol^{-1} \cdot s^{-1}$)	HL_{water} (h)	HL_{soil} (h)
HFIPA	168.04	-2.86 [A]	1.61 [A]	1.742 [B]	1440 [C]	2880 [C]
HF2OH	184.04	-4.74 [D]	0.88 [E]	2.800 [B]	4320 [C]	8640 [C]

[A] EAS-E Suite, IFSQSARv1.1.1 logKow-ppLFER

[B] EPI Web v4.1, AOPWIN_v1.93, the overall OH Rate Constant

[C] EPI Web v4.1, BIOWIN_Ver.4.11 EPI Suite, the Half-Life in Water/Soil

[D] EAS-E Suite, IFSQSARv1.1.1 logKaw-ppLFER/OPERA KAW-Converted from HLC/HenryWin_v3.21 LogKAW-Bond_method (consensus of 3 values)

[E] EAS-E Suite, KOWWIN_v1.69-AFC/IFSQSARv1.1.1 logKow-ppLFER/OPERA LogP-KNN (consensus of 3 values)

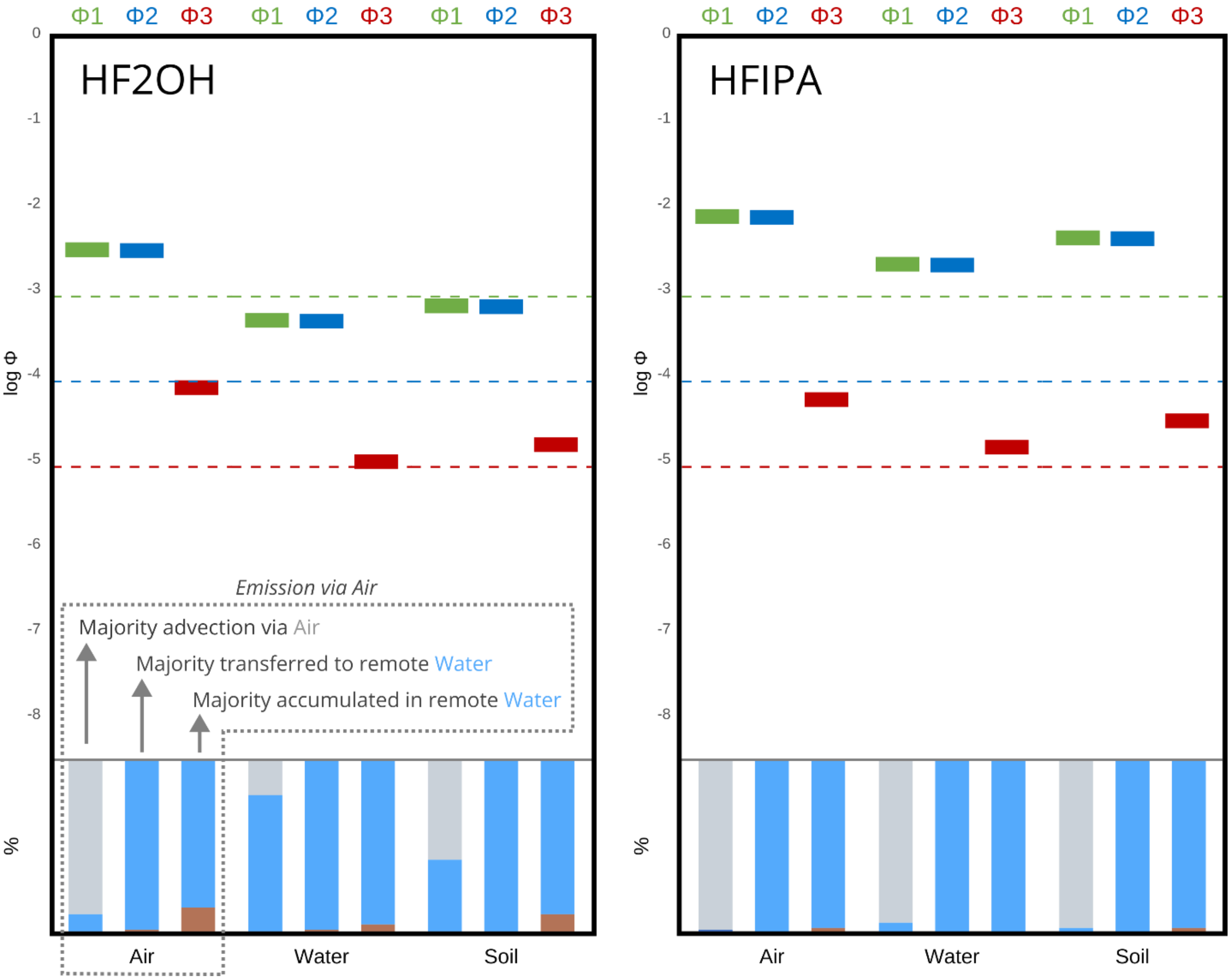

Figure S5. Plotted LRTP screening tool results HF2OH and HFIPA. Labelled POP-like thresholds are shown as dotted lines for the fraction dispersed from the source (ϕ1), the fraction transferred to a remote surface (ϕ2), and the fraction which accumulates in remote media (ϕ3). Differences between sequential logϕ values correspond to fractions dispersed but not transferred to a remote surface (ϕ1 – ϕ2) and fractions degraded following transfer to a remote surface (ϕ2 – ϕ3). Both HFIPA and HF2OH exceed POP-like thresholds for dispersion, transfer, and accumulation for emissions into air. The fractional bars represent the compartment contribution to advection, portions transferred to remote compartments, and portions accumulated in remote compartments, dependent on emission via air, water, or soil. Grey, blue, and brown represent the compartments of air, water, and soil, respectively.

S8. Snow partitioning calculations

The approach described in Meyer et al. (2008) was used in this work.[7] Briefly, chemical space plots for Arctic snow with a density of 4.0·10^5 g/m^3 , a specific surface area of 0.0125 m^2/g, a 4 % water content, a humic acid concentration of 0.009 µm/mL, and a humic acid density of 1.4 g/cm^3 were determined. The snow-air partitioning coefficient (K_{IA}) was determined using a poly-parameter linear free energy relationship (pp-LFER) from Roth et al. (2004) for T = -6.8 °C and adjusted using calculated ΔH_{IA}.[8] The air-water partition coefficients (K_{AW}) at T = 25 °C for the chemicals of interest were all sourced from EAS-E Suite, IFSQSARv1.1.1 logKaw-ppLFER, except for HF2OH which was sourced as a consensus of 3 values (EAS-E Suite, IFSQSARv1.1.1/OPERA KAW-Converted from HLC/HenryWin_v3.21 LogKAW-Bond_method). K_{AW} was adjusted to T = 0 °C for all chemicals using a water-air bulk phase partitioning pp-LFER taken from the UFZ-LSER database and the solute descriptors in Table S17.[9,10] The humic acid-water partition coefficients ($K_{HA/W}$) were calculated using pp-LFERs at multiple temperatures taken from Niederer et al. (2006)[11] and extrapolated to T = 0 °C using the solute descriptors in Table S17. The precursor chemicals investigated include hexafluoroiso-butylene (HFIB), 2,3,3,3-tetrafluoropropene (HFO-1234yf), hexafluoroacetone (HFA), 1,1,1,3,3,3-hexafluoropropane (HFC-236fa), and hexafluoropropylene oxide (HFPO).

Table S17. Solute descriptors and enthalpies used in the snow partitioning calculations.

Chemical	E [A]	S [A]	A [A]	B [A]	L [A]	V [A]	ΔH_{IA} [B]
HFIPA	-0.24	0.55	0.77	0.1	1.392	0.6962	-57.77
HF2OH	-0.005	0.54	1.194	0.78	2.01	0.755	-120.73
HFIB	-0.563	0	0	0	0.11	0.735	-9.94
HFO-1234yf	-0.317	0	0	0	0.32	0.559	-11.49
HFA	-0.576	0.42	0	0.28	0.31	0.653	-28.12
HFC-236fa	-0.59	0.13	0.13	0.02	0.746	0.6375	-21.31
HFPO	-0.318	-0.2	0	0.12	0.91	0.588	-23.02

[A] EAS-E Suite, IFSQSARv1.1.1 - updated for PFAS
[B] in kJ/mol, calculated using eq. 8 in Lei and Wania (2004)[12] based on the pp-LFER by Roth et al. (2002)[13] and an empirical relationship by Goss and Schwarzenbach (1999)[14]

Table S18. Physical-chemical properties at 0 °C for the chemicals displayed on partition plots.

Chemical	LogK_{IA} (/m)	LogK_{AW}	Log$K_{HA/W}$
HFIPA	-2.90	-1.92	1.24
HF2OH	1.29	-3.17	-0.70
HFIB	-6.78	2.75	1.59
HFO-1234yf	-6.65	2.16	1.21
HFA	-5.71	0.44	0.24
HFC-236fa	-5.85	1.42	1.40
HFPO	-5.86	1.59	1.17

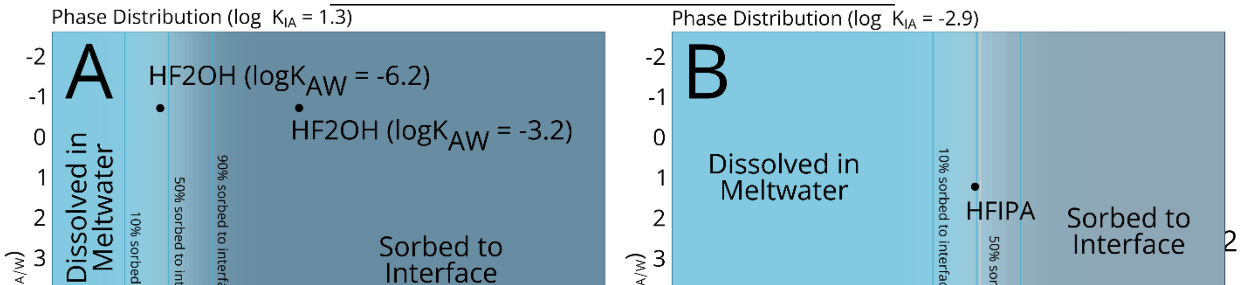

S9. Correlation of air concentrations to snowmelt events

For the time periods of interest, data on daily soil temperature, albedo, and downwelling shortwave radiation were collected using the National Oceanic and Atmospheric Administration's Broadband Radiation Downwelling & Meteorological Scaffold/Tower, collocated with the HiVol PS-1 Air Sampler used in this work, at the Global Atmospheric Watch Laboratory at Alert, NU, Canada. The data was sourced from the NOAA Physical Sciences Laboratory FTP server, accessible at: https://downloads.psl.noaa.gov/psd3/arctic/alert/.

All datasets were downloaded spanning 2014 to 2020 on December 1st 2024. The soil temperature at a depth of 5 cm (5CMTsoil) was used to validate the estimate of the end of the melt period from the Albedo measurements, with the end of the snowmelt defined as an increase in 5CMTsoil to greater or equal to 0 °C. The maximum 5CMTsoil temperature per day was used from the dataset. Albedo was used to determine the start and end of the snow melt events. The start of the melt was defined as the start date of a continuous decrease in albedo reaching 0.6 and the end of the snow melt was defined as the date the albedo reached 0.3.[15] An example albedo plot along with start and end melt times for the periods under investigation are shown in Figure S7 for 2018. Downwelling shortwave radiation (W/m^2) was averaged daily, and the average value across each sampling date range. Downwelling shortwave radiation was used to differentiate samples by collection in polar night and midnight sun. Air temperature corresponding to each air sample collected was calculated as the average air temperature between the start of sample collection and the end of sample collection. Table S19 displays the date ranges of yearly snow melt periods for 2014 to 2020. For the years of 2021-2023, only average air temperature was used to estimate melt periods.

Table S19. Determined melt periods for the years under study. No albedo data was available during 2019, and grouping was completed using soil and air temperature measurements alone. *Melt windows were determined based solely on average air temperature data for 2021-2023.

Year	Melt Start Date	Melt Start Week	Melt End Date	Melt End Week	Date of snow-free soil
2014	June 30, 2014	27	July 16, 2014	29	July 12, 2014
2015	June 11, 2015	25	June 24, 2015	26	June 21, 2015
2016	June 9, 2016	24	June 15, 2016	24	June 12, 2016
2017	June 14, 2017	25	June 24, 2017	26	June 22, 2017
2018	June 19, 2018	25	July 7, 2018	27	July 6, 2018
2019	No albedo data available				June 17, 2019
2020	June 13, 2020	25	June 20, 2020	25	June 19, 2020
2021*		23		25	
2022*		23		25	
2023*		23		25	

Melt samples were grouped based on two criteria: (1) their collection times overlapping with the melt windows shown in Table S19, and (2) the earliest air samples collected when average air

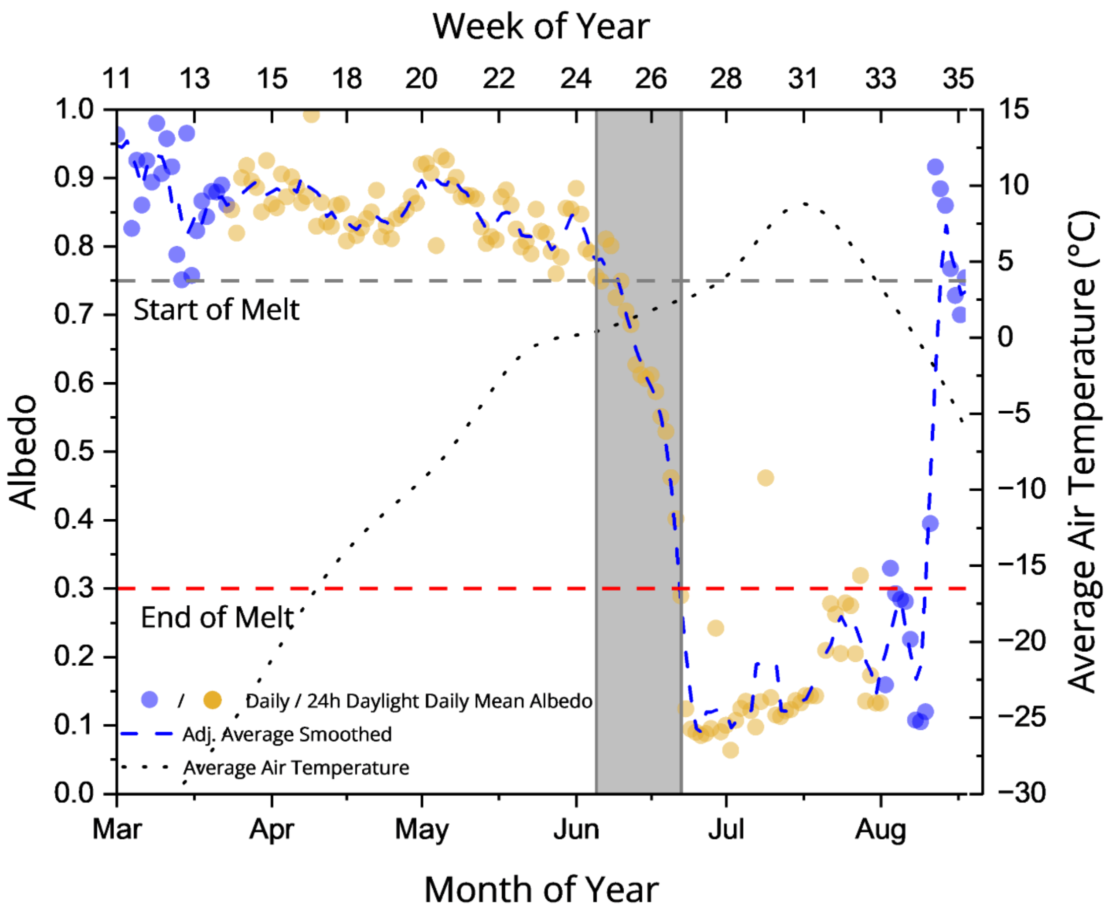

Figure S7. Snow melt sample grouping demonstrated for 2018. Albedo data for years 2014-2020 was averaged daily (with points in yellow symbolizing the 24h daylight period), and 5-adjacent points were averaged to provide a fitted dashed curve. Average air temperature (on per sample basis) plotted as a dotted line. The start of melt was defined as the date albedo decreased continuously to below 0.6, with the end of melt defined as albedo < 0.3. For additional validation, 5 cm deep soil temperature data was used to confirm the end of melt date (defined as T > 0 °C). For 2018, the melt period is outlined in grey. Air concentrations for all compounds over 2014-2020 were grouped into premelt, melt, and postmelt categories. Only samples collected during the 24h daylight period were eligible for grouping into the premelt and postmelt categories.

temperatures exceeded 270 K. Pre-Melt samples were defined as those collected during the 24-hour sunlight period from early April until the start of the Melt period.

Table S20. Spearman correlations between high detection frequency chemicals and 1/T.

	1/T	HFIPA	HF2OH	TFA	PFPrA	PFBA	PFHxA	PFOA	PFNA	PFOS	PFDA
Spearman Corr.	1	0.71684	-0.45705	-0.65301	0.07788	-0.10598	0.0646	-0.21865	-0.03203	0.01262	0.06457
p-value	--	**<0.0001**	**<0.0001**	**<0.0001**	0.29337	0.14138	5.21E-01	0.01026	0.73397	0.89831	0.50273

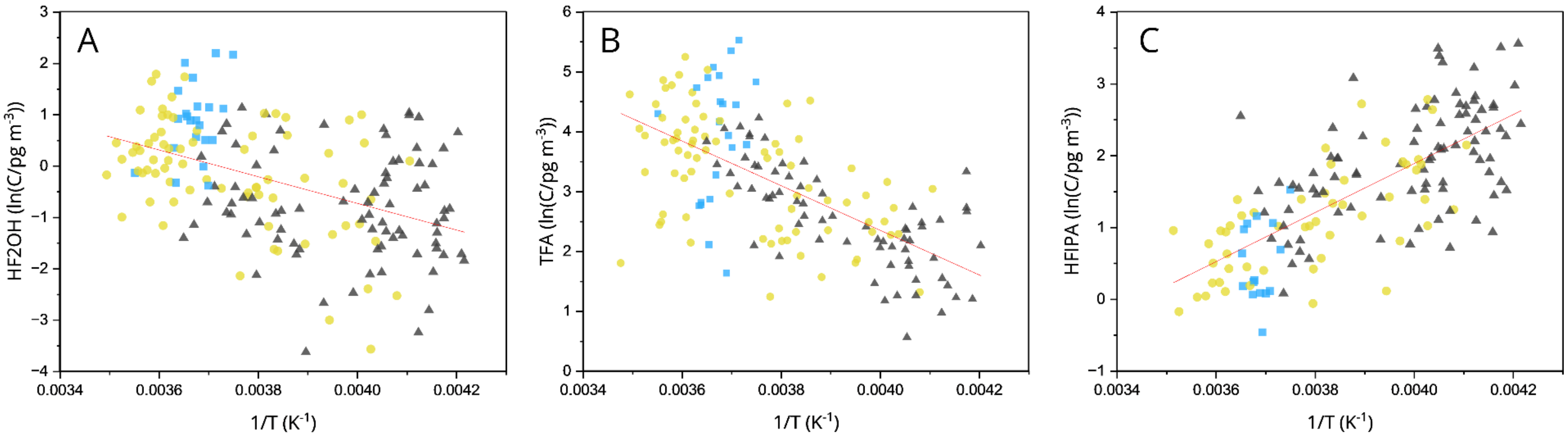

Figure S8. Correlations between atmospheric concentration (ln C, pg/m^3) and inverse temperature (1/T, K^{-1}) for HF2OH (A), TFA (B), and HFIPA (C). Yellow circular points represent samples collected during 24-hour daylight periods, blue square points indicate samples from melt periods, and black triangular points show samples from the remainder of the year. Spearman correlation coefficients are provided in Table S20.

Table S21. Results of Kruskal-Wallis ANOVA for Pre-Melt, Melt, and Post-Melt samples. Dunn's comparison testing was only completed on statistically significant compounds with $p < 0.001$.

Compound	Chi-squared	p-value
HFIPA	23.29	*8.77E-06*
HF2OH	26.04	*2.21E-06*
TFA	27.06	*1.33E-06*
PFPrA	1.20	0.548712
PFBA	5.68	0.05831
PFPeA	12.37	0.002061
PFBuS	1.17	0.557993
PFHxA	4.21	0.121651
PFHpA	3.78	0.150804
PFHxS	2.50	0.286505
PFOA	7.14	0.028199
PFNA	12.69	0.001752
PFOS	0.89	0.639476
PFDA	2.22	0.329309
PFUnDA	5.48	0.064518
PFDS	1.00	0.317311
PFDoDA	7.06	0.029282
PFTrDA	2.40	0.121335
PFTeDA	0.86	0.651439
PFHxDA	1.00	0.317311
PFODA	3.10	0.212754

Table S22. Results of posthoc Dunn's test for compounds with significant differences between Pre-Melt, Melt, and Post-Melt groups.

Compound	Comparison	Z-statistic	P-adjusted
HFIPA	Melt - PostMelt	-0.12	1
HFIPA	Melt - PreMelt	-3.86	**0.0002**
HFIPA	PostMelt - PreMelt	-4.09	**0.0001**
HF2OH	Melt - PostMelt	2.46	0.0209
HF2OH	Melt - PreMelt	5.03	**0.000001**
HF2OH	PostMelt - PreMelt	3.07	0.0032
TFA	Melt - PostMelt	0.48	0.9425
TFA	Melt - PreMelt	4.32	**0.00002**
TFA	PostMelt - PreMelt	4.55	**0.00001**

S10. Trend analysis details and results

The same approach to trend analysis as described in Wong et al. (2021) and Wong et al. (2018) was used.[1,16] In this study, a short-term cutoff period (seasonal harmonic cycle) of 6 months and a long-term cutoff period of 48 months were used. Trend comparisons should be taken with caution by the reader, as they serve to compare the relative rates of decline and increase in concentrations between chemicals. Only data points above MDL were subject to trend analysis.

Table S23. Estimated apparent first order doubling times (t_2, years) for high detection frequency PFAS at Alert. Positive t_2 indicates an increasing trend. A negative t_2 indicates a decreasing trend. The dash sign (-) indicates no data due to no trend.

	PFOA		PFOS		PFBA	
**Period**	**t_2**	**R^2**	**t_2**	**R^2**	**t_2**	**R^2**
2006-2013	2	0.83	1.9	0.96	1.6	0.98
2013-2023	-	-	-2.9	0.69	-	-
2017-2023	7.3	0.86			4	0.92

Table S24. Estimated apparent first order half-lives (t_2, years) for high detection frequency scPFAS at Alert. The dash sign (-) indicates no data due to no trend.

	TFA		PFPrA	
**Period**	**t_2**	**R^2**	**t_2**	**R^2**
2014-2023	-	-	9.9	0.61

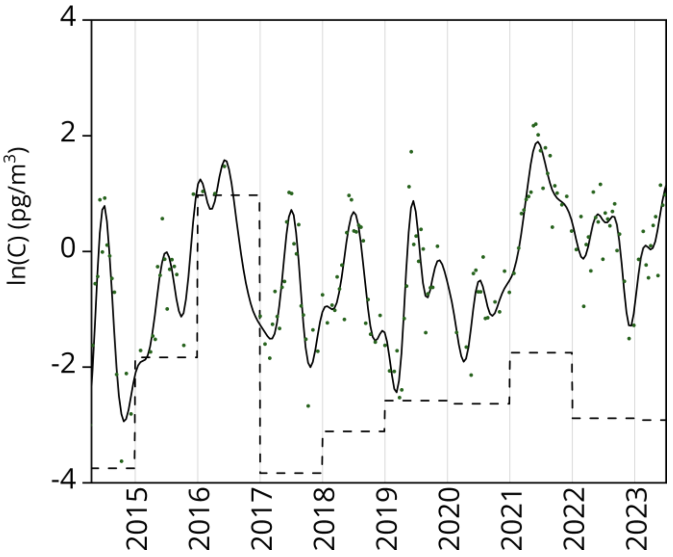

Figure S9. Plotted HFIPA air concentrations. Dashed lines represent MDLs while black lines represent short-term seasonality. Long term trends were not determined due to the semi-quantitative analytical figures of merit for this compound.

S11. References

(1) Wong, F.; Shoeib, M.; Katsoyiannis, A.; Eckhardt, S.; Stohl, A.; Bohlin-Nizzetto, P.; Li, H.; Fellin, P.; Su, Y.; Hung, H. Assessing Temporal Trends and Source Regions of Per- and Polyfluoroalkyl Substances (PFASs) in Air under the Arctic Monitoring and Assessment Programme (AMAP). *Atmos. Environ.* **2018**, *172*, 65–73. https://doi.org/10.1016/j.atmosenv.2017.10.028.

(2) Ye, R.; Di Lorenzo, R. A.; Clouthier, J. T.; Young, C. J.; VandenBoer, T. C. A Rapid Derivatization for Quantitation of Perfluorinated Carboxylic Acids from Aqueous Matrices by Gas Chromatography–Mass Spectrometry. *Anal. Chem.* **2023**, *95* (19), 7648–7655. https://doi.org/10.1021/acs.analchem.3c00593.

(3) Björnsdotter, M. K.; Hartz, W. F.; Kallenborn, R.; Ericson Jogsten, I.; Humby, J. D.; Kärrman, A.; Yeung, L. W. Y. Levels and Seasonal Trends of C1–C4 Perfluoroalkyl Acids and the Discovery of Trifluoromethane Sulfonic Acid in Surface Snow in the Arctic. *Environ. Sci. Technol.* **2021**, *55* (23), 15853–15861. https://doi.org/10.1021/acs.est.1c04776.

(4) Hartz, W. F.; Björnsdotter, M. K.; Yeung, L. W. Y.; Humby, J. D.; Eckhardt, S.; Evangeliou, N.; Ericson Jogsten, I.; Kärrman, A.; Kallenborn, R. Sources and Seasonal Variations of Per- and Polyfluoroalkyl Substances (PFAS) in Surface Snow in the Arctic. *Environ. Sci. Technol.* **2024**, *58* (49), 21817–21828. https://doi.org/10.1021/acs.est.4c08854.

(5) Neuwald, I. J.; Hübner, D.; Wiegand, H. L.; Valkov, V.; Borchers, U.; Nödler, K.; Scheurer, M.; Hale, S. E.; Arp, H. P. H.; Zahn, D. Ultra-Short-Chain PFASs in the Sources of German Drinking Water: Prevalent, Overlooked, Difficult to Remove, and Unregulated. *Environ. Sci. Technol.* **2022**, *56* (10), 6380–6390. https://doi.org/10.1021/acs.est.1c07949.

(6) Breivik, K.; McLachlan, M. S.; Wania, F. The Emissions Fractions Approach to Assessing the Long-Range Transport Potential of Organic Chemicals. *Environ. Sci. Technol.* **2022**, *56* (17), 11983–11990. https://doi.org/10.1021/acs.est.2c03047.

(7) Meyer, T.; Lei, Y. D.; Muradi, I.; Wania, F. Organic Contaminant Release from Melting Snow. 1. Influence of Chemical Partitioning. *Environ. Sci. Technol.* **2009**, *43* (3), 657–662. https://doi.org/10.1021/es8020217.

(8) Roth, C. M.; Goss, K.-U.; Schwarzenbach, R. P. Sorption of Diverse Organic Vapors to Snow. *Environ. Sci. Technol.* **2004**, *38* (15), 4078–4084. https://doi.org/10.1021/es0350684.

(9) Ulrich, N.; E., S.;. Brown, T. N.;. Watanabe, N.;. Bronner, G.;. Abraham, M. H.;. Goss, K. U. UFZ-LSER Database v 3.2 [Internet]. **2017**.

(10) Mintz, C.; Burton, K.; Ladlie, T.; Clark, M.; Acree, W. E.; Abraham, M. H. Enthalpy of Solvation Correlations for Gaseous Solutes Dissolved in Dibutyl Ether and Ethyl Acetate. *Thermochimica Acta* **2008**, *470* (1), 67–76. https://doi.org/10.1016/j.tca.2008.02.001.

(11) Niederer, C.; Goss, K.-U.; Schwarzenbach, R. P. Sorption Equilibrium of a Wide Spectrum of Organic Vapors in Leonardite Humic Acid: Modeling of Experimental Data. *Environ. Sci. Technol.* **2006**, *40* (17), 5374–5379. https://doi.org/10.1021/es0602952.

(12) Lei, Y. D.; Wania, F. Is Rain or Snow a More Efficient Scavenger of Organic Chemicals? *Atmos. Environ.* **2004**, *38* (22), 3557–3571. https://doi.org/10.1016/j.atmosenv.2004.03.039.

(13) Roth, C. M.; Goss, K.-U.; Schwarzenbach, R. P. Adsorption of a Diverse Set of Organic Vapors on the Bulk Water Surface. *J. Colloid Interf. Sci.* **2002**, *252* (1), 21–30. https://doi.org/10.1006/jcis.2002.8446.

(14) Goss, K.-U.; Schwarzenbach, R. P. Empirical Prediction of Heats of Vaporization and Heats of Adsorption of Organic Compounds. *Environ. Sci. Technol.* **1999**, *33* (19), 3390–3393. https://doi.org/10.1021/es980812j.

(15) Cox, C. J.; Stone, R. S.; Douglas, D. C.; Stanitski, D. M.; Divoky, G. J.; Dutton, G. S.; Sweeney, C.; George, J. C.; Longenecker, D. U. Drivers and Environmental Responses to the Changing Annual Snow Cycle of Northern Alaska. *Bull. Am. Met. Soc.* **2017**, *98* (12), 2559–2577. https://doi.org/10.1175/BAMS-D-16-0201.1.

(16) Wong, F.; Hung, H.; Dryfhout-Clark, H.; Aas, W.; Bohlin-Nizzetto, P.; Breivik, K.; Mastromonaco, M. N.; Lundén, E. B.; Ólafsdóttir, K.; Sigurðsson, Á.; Vorkamp, K.; Bossi, R.; Skov, H.; Hakola, H.; Barresi, E.; Sverko, E.; Fellin, P.; Li, H.; Vlasenko, A.; Zapevalov, M.; Samsonov, D.; Wilson, S. Time Trends of Persistent Organic Pollutants (POPs) and Chemicals of Emerging Arctic Concern (CEAC) in Arctic Air from 25 Years of Monitoring. *Sci. Total Environ.* **2021**, *775*, 145109. https://doi.org/10.1016/j.scitotenv.2021.145109.
